# Risk Factors Associated with Renal Involvement in Childhood Henoch-Schönlein Purpura: A Meta-Analysis

**DOI:** 10.1371/journal.pone.0167346

**Published:** 2016-11-30

**Authors:** Han Chan, Yan-Ling Tang, Xiao-Hang Lv, Gao-Fu Zhang, Mo Wang, Hai-Ping Yang, Qiu Li

**Affiliations:** Department of Nephrology, Key Laboratory of the Ministry of Education, Children’s Hospital of Chongqing Medical University, Chongqing, People’s Republic of China; Universita degli Studi di Perugia, ITALY

## Abstract

**Background and objective:**

Henoch-Schönlein purpura (HSP) is an important cause of chronic kidney disease in children. This meta-analysis identified risk factors associated with renal involvement in childhood HSP.

**Methods:**

PubMed, Embase, and Web of Science were searched. The quality of all eligible studies was assessed using the Newcastle-Ottawa scale criteria. An analysis of possible risk factors was conducted to report the odds ratio (OR) and weighted mean difference (WMD).

**Results:**

Thirteen studies (2398 children) revealed 20 possible and 13 significant risk factors associated with renal involvement in HSP, with the following meta-analysis estimates of OR and WMD, with 95% confidence intervals: older age (0.90, 0.61–1.19); age > 10 y (3.13, 1.39–7.07); male gender (1.36, 1.07–1.74); abdominal pain (1.94,1.24–3.04); gastrointestinal bleeding (1.86, 1.30–2.65); severe bowel angina (3.38, 1.17–9.80); persistent purpura (4.02, 1.22–13.25); relapse (4.70, 2.42–9.14); WBC > 15 × 10^9^/L (2.42, 1.39–4.22); platelets > 500 × 10^9^/L (2.98, 1.22–7.25); elevated antistreptolysin O (ASO) (2.17, 1.29–3.64); and decreased complement component 3 (C3) (3.13, 1.62–6.05). Factors not significantly associated with renal involvement were: blood pressure; orchitis; elevated C-reactive protein; elevated erythrocyte sedimentation rate (ESR); and elevated serum IgA/IgE or IgG. Arthritis/arthralgia may be a risk factor according to the criteria of the American College of Rheumatology (1.41, 1.01–1.96).

**Conclusion:**

The following are associated with renal involvement in pediatric HSP: male gender; > 10 y old; severe gastrointestinal symptoms (abdominal pain, gastrointestinal bleeding, and severe bowel angina); arthritis/arthralgia; persistent purpura or relapse; WBC > 15 × 10^9^/L; platelets > 500 × 10^9^/L; elevated ASO; and low C3. Relevant clinical interventions for these risk factors may exert positive effects on the prevention of kidney disease during the early stages of HSP. However, the results should be interpreted cautiously due to the limitations of the studies.

## Introduction

Henoch-Schönlein purpura (HSP) is the most common small vessel vasculitis in childhood, with an annual incidence of 10–20 per 100,000. More than 90% of patients are younger than 10 years old [1]. The clinical features of HSP have been well described and are predominantly non-thrombocytopenic purpura, arthritis, abdominal pain, gastrointestinal bleeding, and glomerulonephritis. Numerous investigations suggest that HSP is not a self-limited disease as previously thought and may eventually develop into chronic kidney disease in childhood [2–3]. Long-term prognosis depends mainly on the severity of renal involvement, which may manifest as persistent hematuria, proteinuria, nephritic or nephrotic syndrome, or even renal failure [4]. To prevent or delay end-stage renal disease, identification of early-stage nephritis is crucial.

The risk factors associated with renal involvement in HSP are not well known although epidemiologic and clinical features and some abnormal laboratory findings have been suggested to have a predictive role [5]. In this meta-analysis, we assessed the quality of available evidence regarding risk factors that may predict renal involvement in childhood HSP, and present a summary of our results.

## Methods

### Literature search strategy

The databases PubMed, Embase, and Web of Science were searched for papers published from January 2000 to September 2016, using basic search terms from combined text and Medical Subject Heading (MeSH) terms, including a MeSH search using ‘Purpura, Henoch-Schönlein’ and a keyword search using the word ‘Henoch-Schönlein purpura’, and terms related to risk factors (including a MeSH search using ‘Risk Factors’, and a keyword search using the words ‘risk factors’). This search strategy was modified to fit each database. References from published review articles were reviewed to identify additional relevant studies (S1 Text). Cohort studies or case-control studies that evaluated the risk factors for developing HSP nephritis (HSPN) were included. Titles and abstracts of articles found were screened, and articles of interest were also selected for evaluation of the full article.

### Study selection criteria

Patients were diagnosed with HSP at < 18 years of age, with consideration for the criteria of the American College of Rheumatology (ACR) [6] and definitions of the European League against Rheumatism (EULAR) [7], in which HSPN is defined as the presence of hematuria or proteinuria at two different times within 6 months of HSP onset. Renal involvement was defined as the presence of proteinuria, hematuria, or blood cell casts. The studies in our meta-analysis must include detailed information after the onset of HSP and the length of follow-up time should be increased at least up to 6 months. Thrombocytopenia and systemic lupus erythematosus at the onset were excluded.

### Data collection and data extraction

Studies were selected by 2 independent reviewers according to the predetermined inclusion criteria, and disagreements were resolved by a third reviewer. Case reports, genetic association studies, review articles, comments, meeting abstracts, and editorial comments were excluded. The extracted data included the studies’ characteristics (publication year), design features, participants (e.g., setting, ethnicity, case number, age, male/female ratio, positive cases of HSP and HSPN, factors (e.g., the laboratory predictors), and diagnostic criteria.

### Bias and quality assessments of the included studies

The recommended checklist of the STROBE (Strengthening the Reporting of Observational Studies in Epidemiology) initiative was used to assess the risk of bias of the included studies [8]. A quality assessment of the studies was performed using Newcastle-Ottawa scale, NOS (http://www.ohri.ca/programs/clinical_epidemiology/oxford.asp), under three main categories: a selection of study; comparability of groups; and determination of outcomes [9].

### Statistical analysis

We estimated the odds ratio (OR) with 95% confidence interval (CI) for dichotomous outcomes. A random-effects model was used regardless of heterogeneity. Heterogeneity among trials was tested using the I^2^ test and considered significant at I^2^ > 50% or *P*- value < 0.1. The random effects model was used for the meta-analysis if there was significant heterogeneity. Subgroup analyses were conducted regarding interval levels in the risk factors (e.g., gender), quality of evidence, and diagnosed criteria. Sensitivity analyses were performed to evaluate the effect of each study on the pooled ORs. The presence of publication bias was also evaluated using the Begg’s and Egger’s test [10,11]. All statistical analyses were performed using Stata 12.0 software (Stata Corp, College Station, TX, (USA) [12].

## Results

### Study selection and characteristics

**Fig 1:**
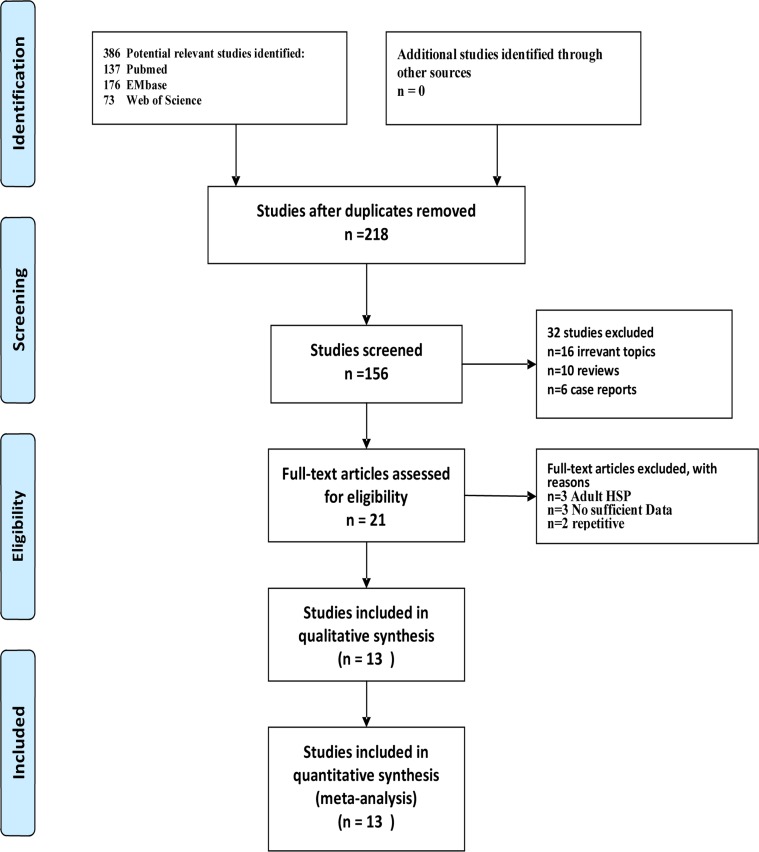
Flowchart of selection process for eligible studies (PRISMA 2009 flow diagram).

Initially, 386 potentially relevant studies were considered (Fig 1), but only 13 studies satisfied the inclusion and exclusion criteria and were included in this meta-analysis (Table 1). These studies were published between 2000 and 2016, and all of them are case-control studies. Two were conducted in Turkey, four in China, and one each in Japan, Korea, Spain, Brazil, Finland, Iran, and Thailand. Studies were performed in 2 major ethnic populations; 8 studies were conducted in Asia (6 East Asian), while 5 studies were conducted with Caucasians. Five studies used the criteria of the EULAR, and 8 used the criteria of the ACR. The 13 studies comprised 2398 children with HSP; 974 of these children had renal involvement.

**Table 1:** Basic characteristics of included studies^a^

	Year	Setting	Ethnicity	HSPN/HSP	Age HSPN/HSP, y	Risk factors ^b^	Criterion
Shin [15]	2006	Korea	Asian	78/128	8.2 ± 3.1/6.7 ± 2.6	1 2 3 4 5 6 7 8 9 10 11 12 14 17	ACR
Jauhola [13]	2015	Finland	Caucasian	102/121	8.2 ± 3.8/6.2 ± 3.0	1 2 4 7 8 13 14 15 16	ACR
Zhao [19]	2015	China	Asian	45/96	7.8 ± 2.6	1 2 3 4 7 10 11 12 13 17	EULAR
Elmas [16]	2014	Turkey	Caucasian	28/79	8.7 ± 4.1/7.1 ± 3.0	1 2 7 9 10 11 13 14 16	EULAR
Mao [20]	2014	China	Asian	267/268	7.5 ± 2.7/6.4 ± 2.5	1 2 7 9 10 11 14 16	EULAR
Anil [17]	2011	Turkey	Caucasian	192/238	7.9 ± 2.8/7.5 ± 2.8	1 2 6 8 9 11 13	ACR
Zhu [21]	2014	China	Asian	80/38	10.41 ± 2.70	1 2 7 8 9 14 15 17	ACR
Mariac [14]	2001	Spain	Caucasian	8/61	6.9 ± 2.2/6.1 ± 3.3	1 2 5 6 7 8 9 13 14	ACR
Kawasaki [18]	2003	Japan	Asian	15/69	9.6 ± 3.7/8.7 ± 3.1	1 4 7 14 16 17	ACR
Limpongsanurak [22]	2011	Thailand	Asian	11/156	6.9 ± 2.6	5 7 12	ACR
Luiz [23]	2007	Brazil	Caucasian	70/72	——	1 2 3 4 5 6 7 14	ACR
Wang [24]	2016	China	Asian	37/34	8.6 ± 2.1	1 2 7 11 14	EULAR
Nickavar [25]	2012	Iran	Caucasian	41/64	7.3 ± 2.6/5.3 ± 2.7	2 5 7	EULAR

^a^ All studies were case-control studies

^b^ Risk factor key: 1, age; 2, gender; 3, persistent purpura; 4, abdominal pain; 5, severe bowel angina; 6, gastrointestinal bleeding; 7, arthritis/arthralgia; 8, relapse; 9, leukocytosis; 10, thrombocytosis; 11, CRP; 12, ASO; 13, ESR; 14, IgA; 15, IgE; 16, IgG; 17, C3.

### Quality of evidence

NOS scale for case-control studies was applied to assess the quality of the evidence. Six studies were judged to be of high relative quality [13–18] and 7 were of medium quality [19–25]. In the selected studies, the controls were not community-based except for Jauhola [13] and Mariac [14]. An additional unclear confounder was not controlled within comparability categories, which may be a possible source of bias. In addition, all of the 7 medium-quality studies may contain bias due to a relatively short-term follow-up. (Table 2)

**Table 2:** Newcastle-Ottawa quality assessment scale (case-control) for studies included in this meta-analysis

		1	2	3	4	5	6	7	8	9	10	11	12	13
Was the case definition adequate	**a.** Yes, with independent validation*; **b.** yes, e.g., record linkage or based on self-reports; **c.** no description	**●**	**●**	**●**	**●**	**●**	**●**	**●**	**●**	**●**	**●**	**●**	**●**	**●**
Representativeness of the case	**a.** Consecutive or obviously representative series of cases*; **b.** potential for selection biases or not stated	**●**	**●**				**●**		**●**	**●**				
Selection of controls	**a.** Community controls*; **b.** hospital controls; **c.** no description		**●**						**●**					
Definition of controls	**a.** No history of disease (endpoint*; **b.** no description of source	**●**	**●**	**●**	**●**	**●**	**●**	**●**	**●**	**●**	**●**	**●**	**●**	**●**
Comparability	**a.** Study controls for_ _ _ _(selecting the most important factor)*; **b.** study controls for any additional factor*	**●**	**●**	**●**	**●**	**●**	**●**	**●**	**●**	**●**	**●**	**●**	**●**	**●**
Ascertainment of exposure	**a.** Secure record (e.g., surgical records)*; **b.** structured interview where blind to case/control status interview not blinded to case/control status*; **d.** written self-report or medical record only; **e.** no description	**●**	**●**	**●**	**●**	**●**	**●**	**●**	**●**	**●**	**●**	**●**	**●**	**●**
Ascertainment for cases & controls	**a.** Yes* **b.** No	**●**	**●**	**●**	**●**	**●**	**●**	**●**	**●**	**●**	**●**	**●**	**●**	**●**
Non-response rate	**a.** Same rate for both groups*; **b.** non-respondents described; **c.** rate different and no designation	**●**	**●**	**●**	**●**	**●**	**●**	**●**	**●**	**●**	**●**	**●**	**●**	**●**
Score		7	8	6	7	6	7	6	8	7	6	6	6	6

*Scored points

The control group of each study was selected from the same population as the case group and was confirmed without kidney damage after the follow-up period. Controls were selected independently by exposure status and without special clinical features. Both groups completed the follow-up time and had similar rates of non-response. To ensure an efficient and valid study, selection and restrict enrollment for patients was conducted in each study. In addition, a stratified analysis (e.g., gender, age, clinical manifestation) and logistic regression for multivariate analysis for confounding factors (except for Limpongsanurak [22] and Nickavar [25]) were conducted in all selected studies. All the analyzed risk factors were shown to be directly linked with renal damage.

### Results of meta-analysis

**Fig 2:**
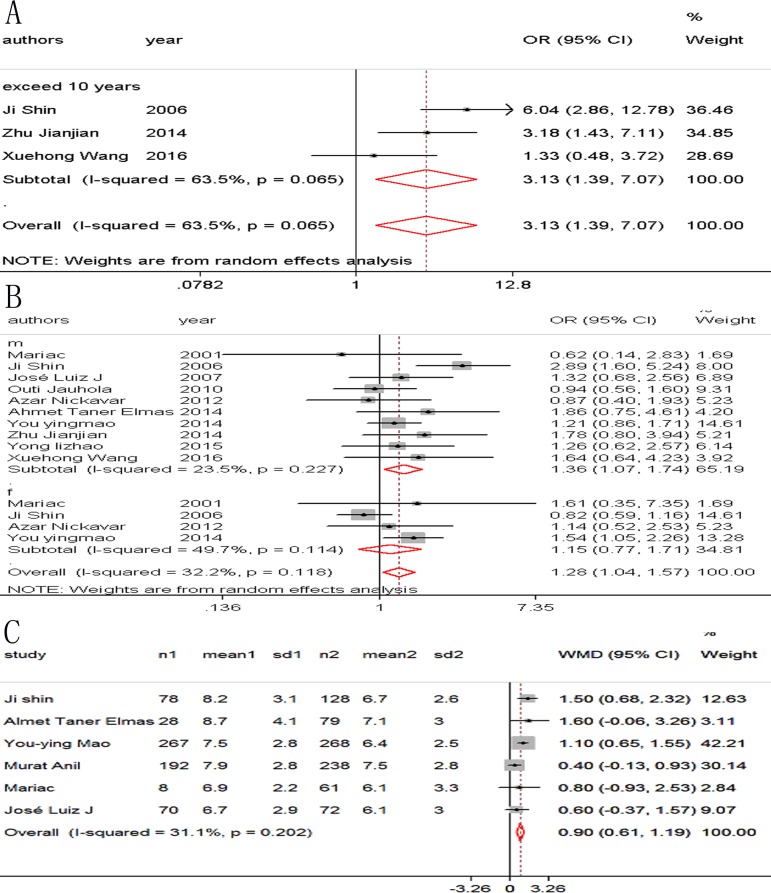
Forest plots of OR/WMD estimates for the following risk factors: (A) age; (B) gender: (C) older age

**Fig 3:**
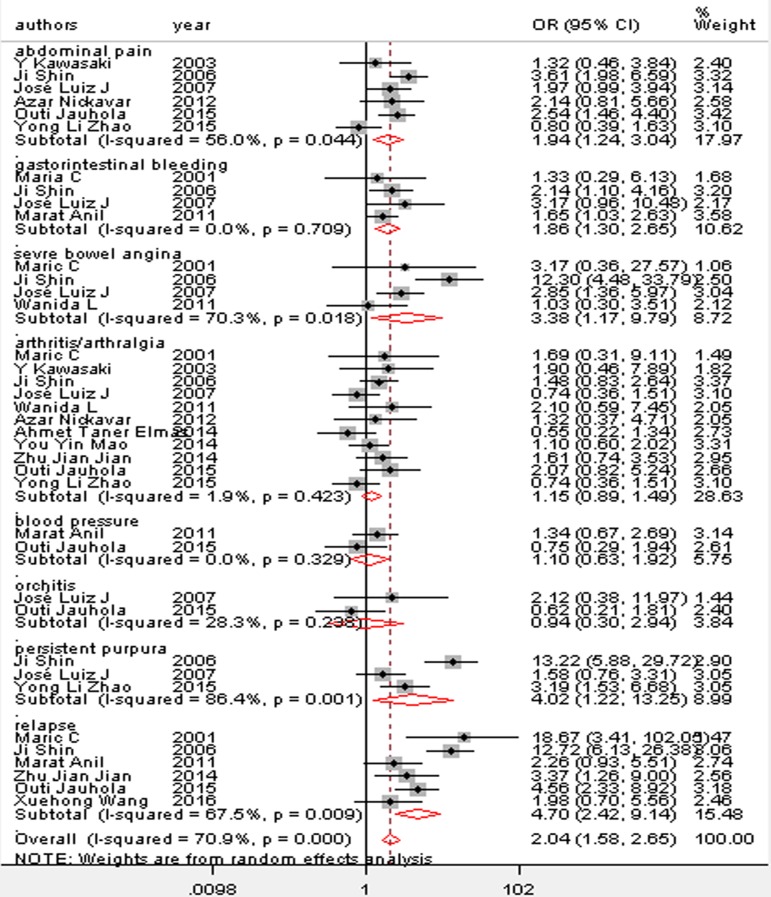
Forest plots of OR estimates for the following risk factors: abdominal pain; gastrointestinal bleeding; severe bowel angina; arthritis or arthralgia; blood pressure; orchitis; persistent purpura; relapse

**Fig 4:**
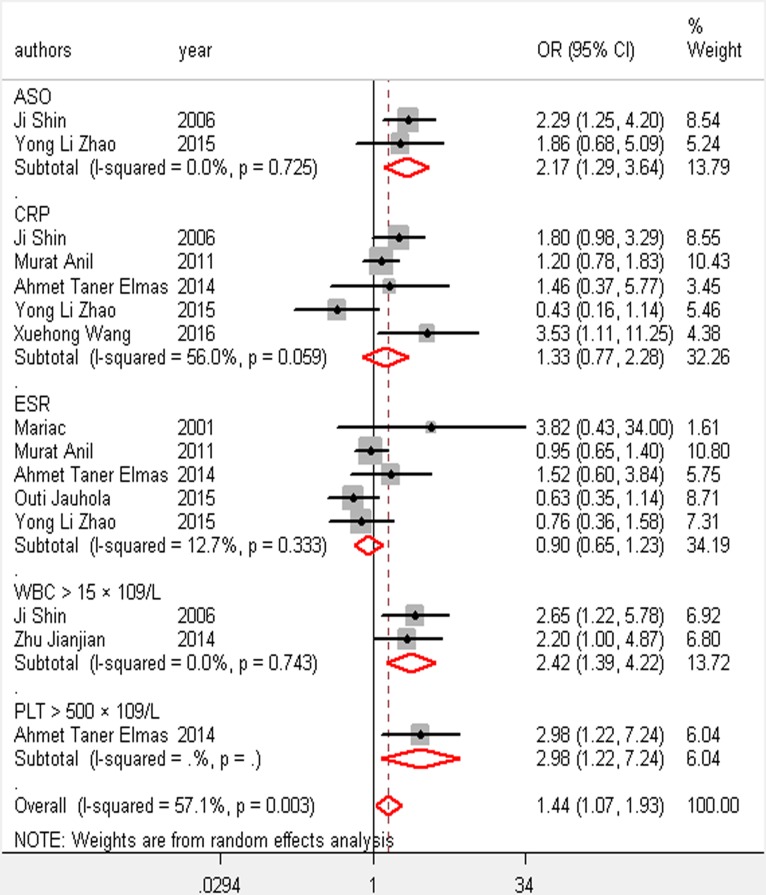
Forest plots of OR estimates for the following risk factors: ASO; CRP; ESR; leukocytosis; thrombocytosis

**Fig 5:**
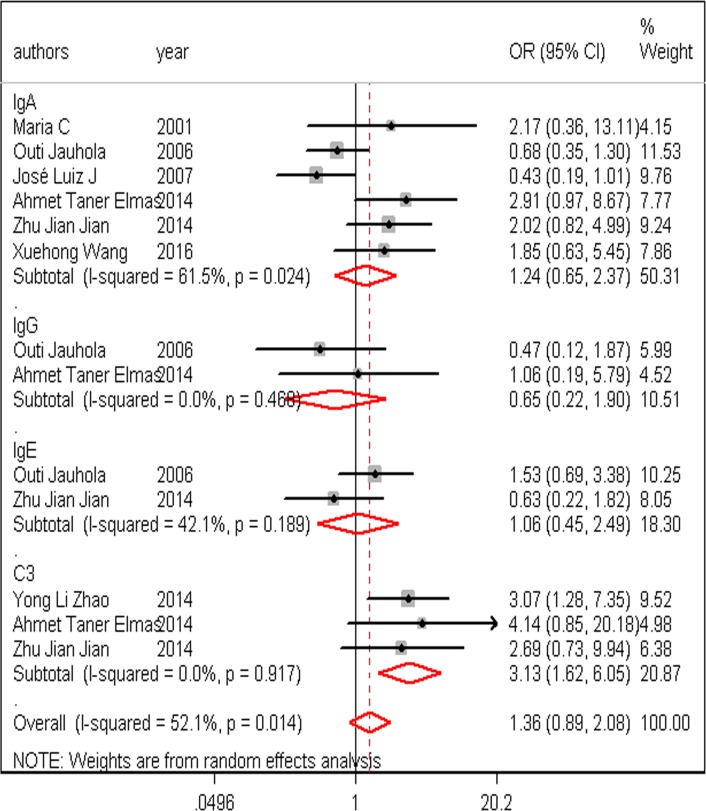
Forest plots of OR estimates for the following risk factors: IgA; IgE; IgG; C3

**Fig 6:**
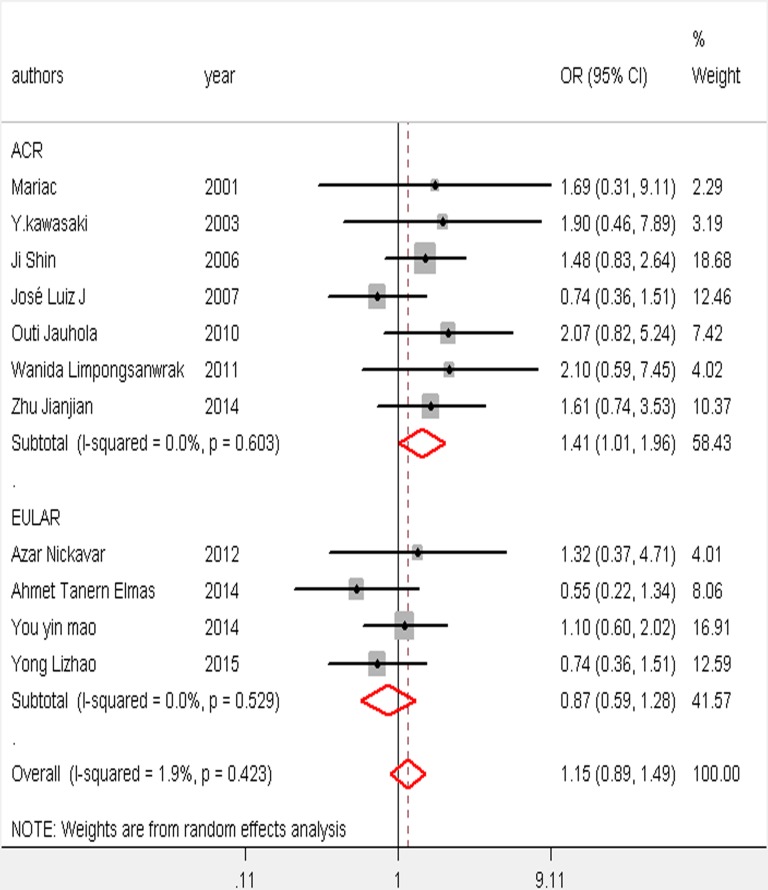
Result of sensitivity analysis and subgroup analysis on arthritis/ arthralgia.

We observed a consistent significant association between 12 risk factors and renal involvement in childhood HSP: gender, age, abdominal pain, gastrointestinal bleeding, severe bowel angina, persistent or relapse purpura, WBC > 15 × 10^9^/L, platelet count > 500 × 10^9^/L, elevated ASO, and decreased complement component 3 (C3; Table 3 and Figs 2–5). In addition, there was no association between girls with HSP and HSPN (OR 1.15; 95% CI [0.77–1.71]) where *P* = 0.12. Arthritis/arthralgia may be a risk factor according to the criteria of the ACR in our analysis (1.41, 1.01–1.96, Fig 6).

**Table 3:** Results of meta-analysis by system

	Possible risk factors	Inconclusive	Results
	Age		Older age WMD = 0.90,95% CI (0.61–1.19), P = 0.00
			>10 year-old = 3.13,95% CI (1.39–7.07), *P* = 0.06
	Boy		OR = 1.36,95% CI (1.07–1.74), *P* = 0.02
		Girl	OR = 1.15,95% CI (0.77–1.71), *P* = 0.15
Digestive tract	Abdominal pain		OR = 1.94,95% CI (1.24–3.04), *P* = 0.04
	Gastrointestinal bleeding		OR = 1.86,95% CI (1.30–2.65), *P* = 0.70
	Severe bowel angina		OR = 3.38,95% CI (1.17–9.80), *P* = 0.02
Skeletal	Arthritis/arthralgia		OR = 1.41,95% CI (1.01–1.96), *P* = 0.60
Dermal	Persistent purpura		OR = 4.02,95% CI (1.22–13.25), *P* = 0.02
	Relapse		OR = 4.70,95% CI (2.42–9.14), *P* = 0.00
		Blood pressure	OR = 1.10,95% CI (0.63–1.92), *P* = 0.42
		Orchitis	OR = 0.94,95% CI (0.30–2.94), *P* = 0.33
Routine blood test	WBC > 15 × 10^9^/L		OR = 2.42,95% CI (1.39–4.22), *P* = 0.00
	PLT > 500 × 10^9^/L		OR = 2.98,95% CI (1.22–7.25), *P* = 0.02
	Positive ASO		OR = 2.17,95% CI (1.29–3.64), *P* = 0.00
		Elevated ESR	OR = 0.90,95% CI (0.65–1.23), *P* = 0.33
		Elevated CRP	OR = 1.33,95% CI (0.77–2.28), *P* = 0.06
Immunologic function		Elevated IgA	OR = 1.24,95% CI (0.65–2.37), *P* = 0.02
		Elevated IgE	OR = 1.06,95% CI (0.45–2.49), *P* = 0.19
		Elevated IgG	OR = 0.65,95% CI (0.22–1.90), *P* = 0.41
	Decreased C3		OR = 3.13,95% CI (1.62–6.05), *P* = 0.91

### Subgroup and sensitivity analysis

**Fig 7:**
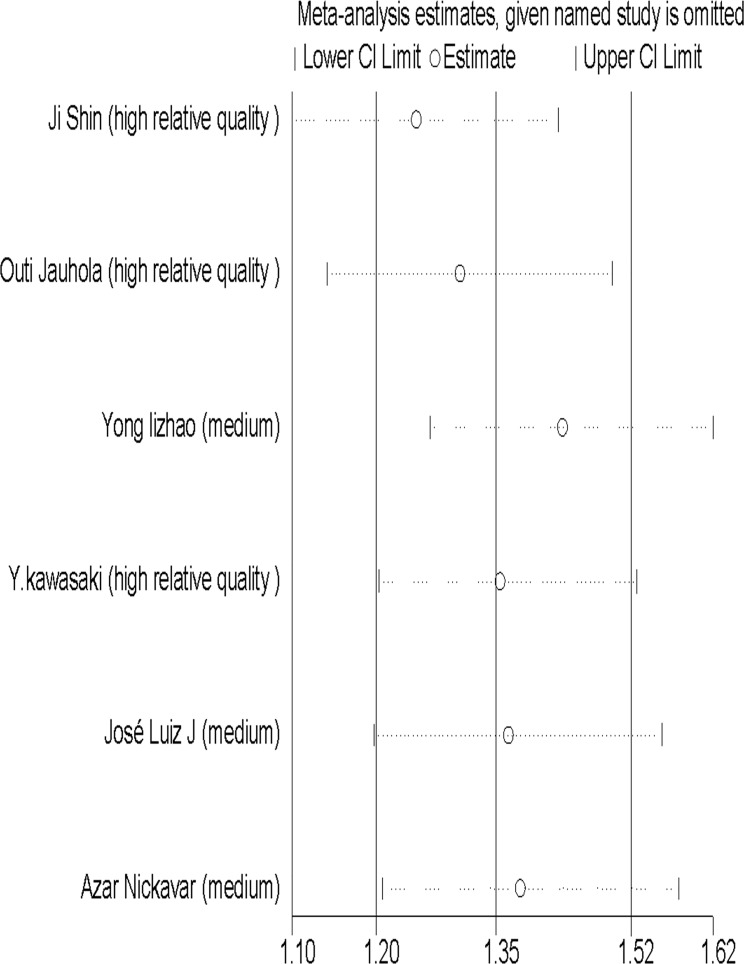
Result of sensitivity analysis on abdominal pain.

**Fig 8:**
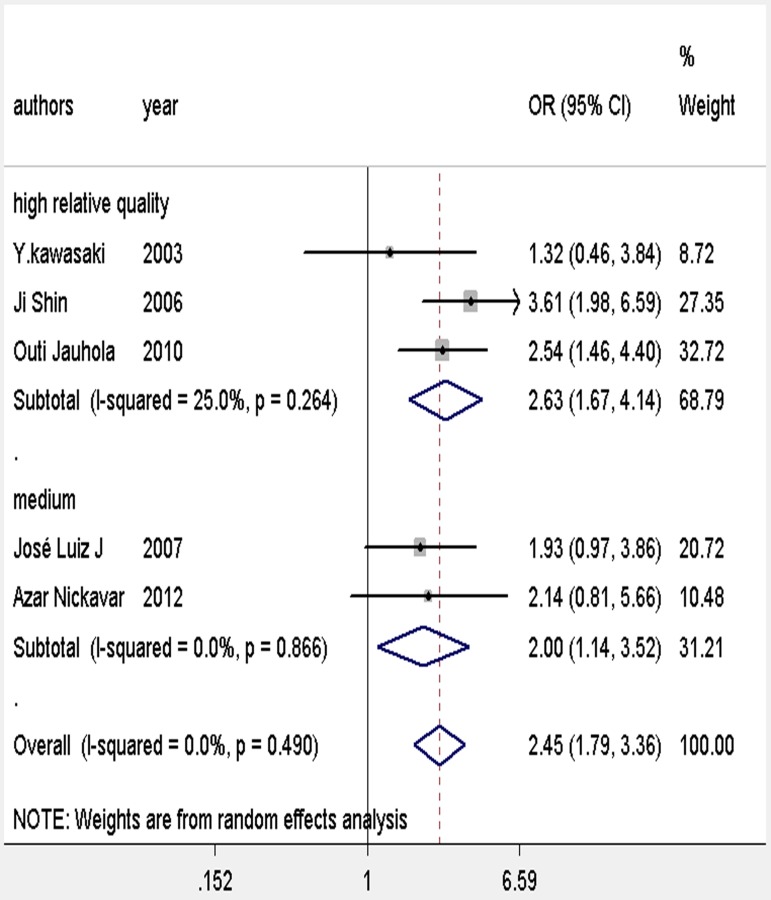
Forest plots of OR estimates for abdominal pain after the deletion of an unstable study.

Except for arthritis/arthralgia, the subgroup analysis showed no evidence of heterogeneity between ACR and EULAR (Fig 6). The sensitivity analysis showed that no individual study significantly altered the results, except for the medium-quality study of Zhao et al. [19], which was the main origin of heterogeneity in abdominal pain (Fig 7). After the deletion of the study, the heterogeneity vanished (Fig 8). A further analysis of studies of different quality revealed that although studies of higher quality may have a more positive effect than medium-quality studies, both had similar results (S2 Appendix). Therefore, the data of each study in the meta-analysis was relatively stable and credible.

### Publication bias

**Fig 9:**
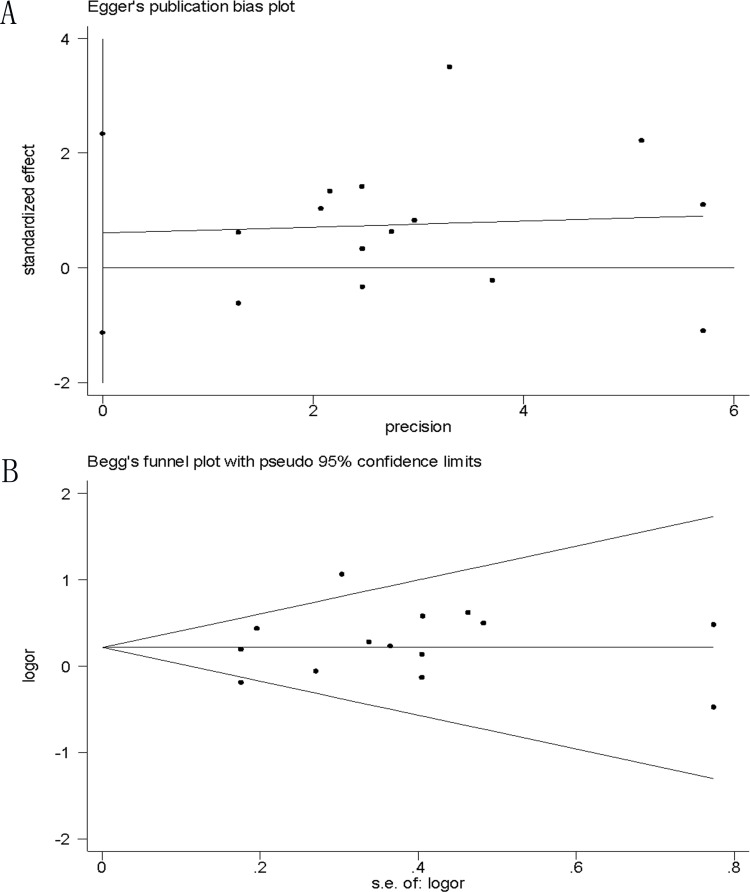
Assessment of Publication bias using (A) Egger’s and (B) Begg’s test.

Assessment of publication bias using Egger’s and Begg’s tests showed that there was no potential publication bias among the included trials (e.g., gender; Egger’s test *P* = 0.46, Begg’s test, *P* = 0.78; Fig 9).

## Discussion

The possible risk factors of renal involvement in childhood Henoch-Schönlein purpura have been reported during past 15 years. Future studies should include a better definition of patients at risk, and then treatment and clinical trials should be adapted to patients’ risk profiles [26]. The present study attempted to determine comprehensively the effect of a multitude of possible risk factors of renal involvement in childhood HSP. The risk factors included demographic features; (age, gender), clinical features; (gastrointestinal tract, skeletal system, skin, testicles, and blood pressure) and some abnormal laboratory findings (such as elevated ASO, elevated ESR, elevated CRP, elevated IgA/IgE/IgG and decreased C3).

A previous meta-analysis was published by Mao et al. [27], which suggested that older age; elevated blood pressure, C3, hemoglobin, and urea nitrogen; and decreased albumin were risk factors for renal damage in HSP patients. In detail, the analysis included 8 studies (12 possible risk factors) and comprised 1222 patients, most of which were experimental studies without follow-up and focused only on laboratory findings in HSP patients. To acquire adequate and reliable clinical literature, our meta-analysis adopted a strict search strategy and included 13 studies (additional 1176 patients and 20 possible risk factors) that concern not only abnormal laboratory findings, but also focus on other vital risk factors (e.g., epidemiological features and clinical manifestations) in children with HSP. Meanwhile, we further assessed heterogeneity by performing subgroup or sensitivity analyses of stratified risk factors (e.g., gender), quality of evidence, and diagnostic criteria of HSP which have a great bearing on the interpretation of the results. In addition, publication bias and potential confounding factors among the included trials were well analyzed in our meta-analysis.

The criteria of the ACR and EULAR were implemented to define HSP. Some studies have adopted the former, while others have utilized the European; Except for arthritis/ arthralgia sensitivity analyses have shown no evidence of heterogeneity observed between both. In fact, all patients with HSP complained of purpura without thrombocytopenia [28]. But while palpable purpura was found in 100% of the cases according to the ACR criteria, the EULAR criteria base a diagnosis of HSP on other symptoms [29].

The majority of patients were diagnosed between the ages of 2 and 10 years_._ Sano et al. [30] concluded that age older than 4 years was an independent risk factor for renal involvement, while according to Jauhola et al. [13] age over 8 years at onset was a risk factor for developing nephritis. Our present meta-analysis showed that older children were at higher risk of renal involvement than younger children. Furthermore, the subgroup analysis revealed that children over 10 years old were more likely to suffer HSPN. Therefore, careful attention should be paid especially to children older than 10 years at onset (Table 3 and Fig 2).

Our meta-analysis based on 10 studies showed that boys are at higher risk for renal involvement than girls. This result is similar to a previous epidemiological survey based on 1.1 million children younger than 17 years in West Midland which reported a boy-to-girl ratio of 1.2:1.0 [31].Male predominance has also been reported in other studies [16, 32, 33]. Male hormones may have a pathogenic role in the course of HSP [34], but other studies reported that HSPN was more common in girls [14, 23] (Table 3 and Fig 2).

Digestive tract symptoms (abdominal pain, gastrointestinal bleeding, and severe bowel angina) and skeletal system symptoms (arthritis/arthralgia) are the predominant manifestations in HSP. The meta-analysis based on 4 studies showed that gastrointestinal bleeding also increased the risk of renal involvement. Even so, few published articles have recognized gastrointestinal bleeding as a risk factor in HSP, and more cohort studies are necessary to identify whether gastrointestinal bleeding is an independent risk factor of HSPN(Table 3 and Fig 3).

Our review showed that digestive tract symptoms (abdominal pain, gastrointestinal bleeding, and severe bowel angina) were strongly related to renal involvement. In a retrospective study of 141 patients with HSP, Zhao et al. [19] showed that abdominal pain was not related to HSPN. However, 45% of the patients were complicated with obesity and 29.8% of them had a long disease course. This was a distinctly different epidemiological feature from other studies, and the possibility of admission bias could explain the heterogeneity (Fig 7). Abdominal pain and severe bowel angina are both unpleasant when patients suffer gastrointestinal involvement in HSP. Our analysis suggested that the two levels of gastrointestinal reactions are essentially equivalent for predicting renal injury in HSP. The mechanism probably is due to early systemic corticosteroid administration for severe abdominal pain, which did not prevent severe renal involvement [35]. As gastrointestinal symptoms are easily missed in clinical practice, more attention should be paid to these patients (Table 3 and Fig 3).

The sensitivity analysis showed a completely different result regarding arthritis/arthralgia between the criteria of the ACR and EULAR and the difference between two criteria may help to explain. For skeletal system symptoms (arthritis/arthralgia), a relatively independent type of HSP according to the criteria of the EULAR, patients with arthritis/arthralgia onset were assigned equally to the HSP or HSPN group. This distribution to compare these groups would not be possible if the criteria of the ACR were adopted (Table 3 and Fig 3).

Most identified studies showed an important association between abnormal skin manifestations (persistent purpura or relapse) and HSPN [36]. According to our meta-analysis, the risk of renal damage in HSP children with persistent purpura is 1.22–13.25-fold that of patients without persistent purpura, and the incidence of purpura relapse with renal involvement is 2.7-11-fold that of those who had no relapse. The study of Rigante et al. [37] showed that only persistent purpura for more than one month was a risk of renal involvement in HSPN. The mechanism probably is due to persistent small vascular inflammation and immune complex deposition, which triggers a series of inflammatory reactions that may damage kidney tissues (Table 3 and Fig 3).

Our analysis showed that WBC >15 × 10^9^/L and platelet count >500 × 10^9^/L were highly correlated with renal involvement in HSP, and a WBC of 10 × 10^9^/L to 15 × 10^9^/L has no statistical link to renal involvement. The mechanism may be a tissue injury induced by inflammatory agents secreted by neutrophils triggered by an inflammation-induced factor [37]. Anti-neutrophil cytoplasmic antibody was found to predict renal damage induced by HSP [38]. Thrombocytosis may be an early and useful indicator, and early studies have suggested that reducing the platelet count may be an effective method to avoid renal involvement [39, 40]. However, a systematic review and meta-analysis of 13 randomized controlled trials performed by Hahn et al. [41] revealed that there is no benefit in giving antiplatelet agents, such as dipyridamole and aspirin, to prevent kidney disease. Heparin may be effective but it is potentially dangerous and should be taken cautiously. Additionally, there are no evidence that prednisone is of benefit in preventing renal damage in HSP according to Hahn et al. [41] and Chartapisak et al. [42] (Table 3 and Fig 4).

ASO is an antibody specific to streptolysin, which indicates streptococcal infections. Most patients also had a history of upper respiratory tract infection [43] and a large proportion was infected with streptococci. Our analysis suggested that upper respiratory tract infection, especially streptococcal infection related to high ASO, precedes HSPN [44]. So far, the mechanism underlying an association between elevated upper respiratory tract infection with streptococcus and kidney damage in HSP is unknown. According to the analysis, elevated ESR and CRP are not related to HSPN (Table 3 and Fig 4).

From the reviewed literature, some patients with HSPN had low C3 (10%-20%), elevated IgA (20%-50%), or both [31]. Our analysis also revealed that low C3 is an independent risk factor in HSP, which is a feature of post-streptococcal glomerulonephritis. Fretzayas et al. [45] reported elevated IgA in 73% of all patients with HSP and in 95% of patients with HSPN, but latter studies did not confirm elevated IgA as a risk factor associated with HSPN. In our present analysis, elevated IgG and IgE may have no connection with HSPN. There were only two papers that reported a link between serum IgG and IgE and HSPN, but the data for our analysis was not sufficient to calculate the OR and obtain a reliable result (Table 3 and Fig 5).

Although some studies have claimed that high blood pressure and orchitis could be risk factors in HSP children, our meta-analysis showed that blood pressure and orchitis did not predict the occurrence of renal involvement (Table 3 and Fig 3).

In the meta-analysis, neither renal failure nor end stage renal disease were detected or described in the selected studies. HSP is more common among children than adults, but there are more end-stage renal disease events in adults [46]. However, management of HSP is difficult for pediatricians because of insufficient clinical data in adult stage. Previous studies reported that severity and persistence of proteinuria were predictive of eventual renal failure [47,48] while Butani [49] and Sanders and Wyatt [50]suggested that there are no clinical features or prognostic markers to reliably identify children at highest risk for disease progression. Limited numbers of patients and appropriate studies at presentation increase the difficulty in performing a meta-analysis of a link between HSPN and end stage renal disease. Therefore, a long-term follow-up should be conducted for children with HSPN to determine prognosis when there is renal involvement.

Early identification of the risk factors of renal involvement in HSP has important implications for intervention and management. A sensitive scoring system based on these risk factors at onset to predict HSPN may become a focus of research. For children with gastrointestinal tract symptoms at onset, early gastrointestinal endoscopy is a good tool for assisting diagnosis of HSP [51]. Children with more than one risk factor require more intervention than those who are without those risk factors. The effectiveness of relevant preventive actions has been confirmed: A clinical trial reported that leukocytapheresis could not only ameliorate gastrointestinal symptoms, but also prevent HSPN, persistent purpura, and relapse in HSP. For persistent purpura and relapse, cyproheptadine could help prevent or at least decrease the occurrence of HSPN in children [52].

Some shortcomings of the present meta-analysis should be considered. Firstly, our analysis was based only on case-control studies, and the above risk factors cannot be relied on as causal factors of renal involvement in HSP. The risk factors of renal injury in HSP remain unclear. Case reports that point to new risk factors are increasing every year, and these may be a source of confounding factors in the included studies. Secondly, measurements and missing data were not described in detail in most of the included studies, which may indicate a possible bias. Thirdly, grey literature was not considered, and only 2 major ethnic populations was included, which may result in reporting and ethnicity selection bias. Fourthly, although there is no statistical or clinical heterogeneity across the case-control studies, potential bias still exists because of the 2 HSP diagnostic systems. For atypical HSP children, we should take a cautious attitude towards the above risk factors. Finally, for several risk factors (e.g., age,leukocytosis, thrombocytosis, and elevated ASO), relevant literature is limited and verification from clinical data is required.

## Conclusions

In conclusion, our study highlights the following as factors associated with renal involvement in pediatric HSP: male gender, age > 10 years, severe gastrointestinal symptoms (including abdominal pain, gastrointestinal bleeding, and severe bowel angina), arthritis/arthralgia, persistent purpura or relapse, WBC > 15 × 10^9^/L, platelets > 500 × 10^9^/L, elevated ASO, and decreased C3. More attention should be paid to those children who have one or more of the above risk factors, and a sensitive scoring system based on these risk factors at onset may help predict renal injury in HSP patients. Relevant clinical intervention may exert positive effects on the prevention of kidney disease in the early stage of HSP. Well-designed and conventionally reported studies with a considerable number of children with HSP are necessary to determine the possible link between these 13 risk factors and HSPN.

## Supporting Information

S1 Appendix PRISMA 2009 Checklist (DOC) (DOC)

S2 Appendix Subgroup analysis for quality of evidence: (A) age; (B) male gender; (C) older age; (D) abdominal pain; (E) gastrointestinal bleeding; (F) severe bowel angina; (G) arthritis/arthralgia; (H) persistent purpura; (I) relapse; (J) leukocytosis; (K) thrombocytosis; (L) ASO; (M) C3
(ZIP)

S1 Text Search strategy for each database (DOC)

## Abbreviations

ACR: American College of Rheumatology
ASO: antistreptolysin O
C3: complement component 3
CRP: C-reactive protein
ESR: erythrocyte sedimentation rate
EULAR: European League Against Rheumatism
HSP: Henoch-Schönlein purpura
HSPN: Henoch-Schönlein purpura nephritis
OR: odds ratio
WBC: white blood cell
WMD: weighted mean difference
