# Supplementary material for: Risk Factors Associated with Renal Involvement in Childhood Henoch-Schönlein Purpura: A Meta-Analysis
Source: PLoS One. 2016 Nov 30;11(11):e0167346. doi: 10.1371/journal.pone.0167346 (PMC5130272; doi:10.1371/journal.pone.0167346)
Supplement: S1 Appendix — (DOC) [file pone.0167346.s001.doc]

| **Section/topic** | **#** | **Checklist item** | **Reported on page #** |
| --- | --- | --- | --- |
| **TITLE** | | |  |
| Title | 1 | Risk factors associated with renal involvement in childhood Henoch-Schönlein purpura: A meta-analysis | 1 |
| **ABSTRACT** | | |  |
| Structured summary | 2 | **Background and objective**  Henoch-Schönlein purpura (HSP) is an important cause of chronic kidney disease in children. This meta-analysis identified risk factors associated with renal involvement in childhood HSP.  **Methods**  PubMed, Embase, and Web of Science were searched. The quality of all eligible studies was assessed using the Newcastle-Ottawa scale criteria. An analysis of possible risk factors was conducted to report the odds ratio (OR) and weighted mean difference (WMD).  **Results**  Thirteen studies (2398 children) revealed 20 possible and 13 significant risk factors associated with renal involvement in HSP, with the following meta-analysis estimates of OR and WMD, with 95% confidence intervals: older age (0.90, 0.61-1.19); age > 10 y (3.13, 1.39-7.07); male gender (1.36, 1.07-1.74); abdominal pain (1.94,1.24-3.04); gastrointestinal bleeding (1.86, 1.30-2.65); severe bowel angina (3.38, 1.17-9.80); persistent purpura (4.02, 1.22-13.25); relapse (4.70, 2.42-9.14); WBC > 15 × 10^9^/L (2.42, 1.39-4.22); platelets > 500 × 10^9^/L (2.98, 1.22-7.25); elevated antistreptolysin O (ASO) (2.17, 1.29-3.64); and decreased complement component 3 (C3) (3.13, 1.62-6.05). Factors not significantly associated with renal involvement were: blood pressure; orchitis; elevated C-reactive protein; elevated erythrocyte sedimentation rate (ESR); and elevated serum IgA/IgE or IgG. Arthritis/arthralgia may be a risk factor according to the criteria of the American College of Rheumatology (1.41, 1.01-1.96).  **Conclusion**  The following are associated with renal involvement in pediatric HSP: male gender; > 10 y old; severe gastrointestinal symptoms (abdominal pain, gastrointestinal bleeding, and severe bowel angina); arthritis/arthralgia; persistent purpura or relapse; WBC > 15 × 10^9^/L; platelets > 500 × 10^9^/L; elevated ASO; and low C3. Relevant clinical interventions for these risk factors may exert positive effects on the prevention of kidney disease during the early stages of HSP. However, the results should be interpreted cautiously due to the limitations of the studies. | 2-3 |
| **INTRODUCTION** | | |  |
| Rationale | 3 | Henoch-Schönlein purpura (HSP) is the most common small vessel vasculitis in childhood, with an annual incidence of 10-20 per 100,000. | 5 |
| Objectives | 4 | Participants: Patients were diagnosed with HSP at < 18 years of age, with consideration for the criteria of the American College of Rheumatology (ACR) and definitions of the European League Against Rheumatism (EULAR)  Interventions : None  Comparisons: Patients were diagnosed with HSPN  Outcomes: Epidemiologic and clinical features and some abnormal laboratory findings  Study design: Cohort studies or case-control studies | 5 |
| **METHODS** | | |  |
| Protocol and registration | 5 | None | 5 |
| Eligibility criteria | 6 | Patients were diagnosed with HSP at < 18 years of age, with consideration for the criteria of the American College of Rheumatology (ACR)and definitions of the European League Against Rheumatism (EULAR) , in which HSPN is defined as the presence of hematuria or proteinuria at two different times within 6 months of HSP onset. Renal involvement was defined as the presence of proteinuria, hematuria, or blood cell casts. The studies in our meta-analysis must include detailed patient information after the onset of HSP and the length of follow-up time should be increased at least up to 6 months. Thrombocytopenia and Systemic lupus erythematosus at the onset were excluded. | 5 |
| Information sources | 7 | PubMed, Embase, and Web of Science | 5 |
| Search | 8 | The databases PubMed, Embase, and Web of Science were searched for papers published from January 2000 to September 2016, using basic search terms from combined text and Medical Subject Heading (MeSH) terms, including a MeSH search using ‘Purpura, Henoch-Schönlein’ and a keyword search using the word ‘Henoch-Schönlein purpura’, and terms related to risk factors (including a MeSH search using ‘Risk Factors’, and a keyword search using the words ‘risk factors’). This search strategy was modified to fit each database. References from published review articles were reviewed to identify additional relevant studies | 5 |
| Study selection | 9 | Studies were selected by 2 independent reviewers according to the predetermined inclusion criteria, and disagreements were resolved by a third reviewer. Case reports, genetic association studies, review articles, comments, meeting abstracts, and editorial comments were excluded. The extracted data included the studies’ characteristics (publication year), design features, participants (e.g. setting, ethnicity, case number, age, male/female ratio, positive cases of HSP and HSPN, factors (e.g. the laboratory predictors), and diagnostic criteria. | 6 |
| Data collection process | 10 | The extracted data included the studies’ characteristics (publication year), design features, participants (e.g. setting, ethnicity, case number, age, male/female ratio, positive cases of HSP and HSPN, factors (e.g. the laboratory predictors), and diagnostic criteria. | 6 |
| Data items | 11 | The extracted data included the studies’ characteristics (publication year), design features, participants (e.g. setting, ethnicity, case number, age, male/female ratio, positive cases of HSP and HSPN, factors (e.g. the laboratory predictors), and diagnostic criteria. | 5-6 |
| Risk of bias in individual studies | 12 | The recommended checklist of the STROBE (Strengthening the Reporting of Observational Studies in Epidemiology) initiative was used to assess the risk of bias of the included studies . A quality assessment of the studies was performed using Newcastle-Ottawa scale. | 6 |
| Summary measures | 13 | We estimated the odds ratio (OR) with 95% confidence interval (CI) for dichotomous outcomes. A random-effects model was used regardless of heterogeneity. | 6 |
| Synthesis of results | 14 | Subgroup analyses were conducted regarding interval levels in the risk factors (e.g., gender), quality of evidence, and diagnosed criteria. Sensitivity analyses were performed to evaluate the effect of each study on the pooled ORs. The presence of publication bias was also evaluated using the Begg’s and Egger’s test . All statistical analyses were performed using Stata 12.0 software (Stata Corp, College Station, TX, (USA). | 6-7 |

Page 1 of 2

| **Section/topic** | **#** | **Checklist item** | **Reported on page #** |
| --- | --- | --- | --- |
| Risk of bias across studies | 15 | In the selected studies, the controls were not community-based except for Jauhola and Mariac. An additional unclear confounder was not controlled within comparability categories, which may be a possible source of bias. In addition, all of the 7 medium-quality studies may contain bias due to a relatively short-term follow-up. | 10 |
| Additional analyses | 16 | Arthritis/arthralgia may be a risk factor according to the criteria of the ACR in our analysis | 10 |
| **RESULTS** | | |  |
| Study selection | 17 | Initially, 386 potentially relevant studies were considered, but only 13 studies satisfied the inclusion and exclusion criteria and were included in this meta-analysis. | 7 |
| Study characteristics | 18 | These studies were published between 2000 and 2016, and all of them are case-control studies. Two were conducted in Turkey, four in China, and one each in Japan, Korea, Spain, Brazil, Finland, Iran, and Thailand. Studies were performed in 2 major ethnic populations; 8 studies were conducted in Asia (6 East Asian), while 5 studies were conducted with Caucasians. Five studies used the criteria of the EULAR, and 8 used the criteria of the ACR. The 13 studies comprised 2398 children with HSP; 974 of these children had renal involvement. | 7 |
| Risk of bias within studies | 19 | Six studies were judged to be of high relative quality and 7 were of medium quality. | 10 |
| Results of individual studies | 20 | 13 significant risk factors associated with renal involvement in HSP, with the following meta-analysis estimates of OR and WMD, with 95% confidence intervals: older age (0.90, 0.61-1.19); age > 10 y (3.13, 1.39-7.07); male gender (1.36, 1.07-1.74); abdominal pain (1.94,1.24-3.04); gastrointestinal bleeding (1.86, 1.30-2.65); severe bowel angina (3.38, 1.17-9.80); persistent purpura (4.02, 1.22-13.25); relapse (4.70, 2.42-9.14); WBC > 15 × 10^9^/L (2.42, 1.39-4.22); platelets > 500 × 10^9^/L (2.98, 1.22-7.25); elevated antistreptolysin O (ASO) (2.17, 1.29-3.64); and decreased complement component 3 (C3) (3.13, 1.62-6.05). | 13 |
| Synthesis of results | 21 | the following as factors associated with renal involvement in pediatric HSP: male gender, age > 8 years, severe gastrointestinal symptoms (including abdominal pain, gastrointestinal bleeding, and severe bowel angina), arthritis/arthralgia, persistent purpura or relapse, WBC > 15 × 10^9^/L, platelets > 500 × 10^9^/L, elevated ASO, and decreased C3. | 13 |
| Risk of bias across studies | 22 | Assessment of publication bias using Egger’s and Begg’s tests showed that there was no potential publication bias among the included trials (e.g., gender; Egger’s test *P* = 0.46, Begg’s test, *P* = 0.78). | 16 |
| Additional analysis | 23 | The sensitivity analysis showed that no individual study significantly altered the results, except for the medium-quality study of Zhao et al., which was the main origin of heterogeneity in abdominal pain . After the deletion of the study, the heterogeneity vanished. A further analysis of studies of different quality revealed that although studies of higher quality may have a more positive effect than medium-quality studies, both had similar results . Therefore, the data of each study in the meta-analysis was relatively stable and credible. | 16 |
| DISCUSSION | | |  |
| Summary of evidence | 24 | The present study attempted to determine comprehensively the effect of a multitude of possible risk factors of renal involvement in childhood HSP. The risk factors included demographic features; (age, gender), clinical features; (gastrointestinal tract, skeletal system, skin, testicles, and blood pressure) and some abnormal laboratory findings (such as elevated ASO, elevated ESR, elevated CRP, elevated IgA/IgE/IgG and decreased C3). | 16 |
| Limitations | 25 | Firstly, our analysis was based only on case-control studies, and the above risk factors cannot be relied on as causal factors of renal involvement in HSP. The risk factors of renal injury in HSP remain unclear. Case reports that point to new risk factors are increasing every year, and these may be a source of confounding factors in the included studies. Secondly, measurements and missing data were not described in detail in most of the included studies, which may indicate a possible bias. Thirdly, grey literature was not considered, and only 2 major ethnic populations was included, which may result in reporting and ethnicity selection bias. Fourthly, although there is no statistical or clinical heterogeneity across the case-control studies, potential bias still exists because of the 2 HSP diagnostic systems. For atypical HSP children, we should take a cautious attitude towards the above risk factors. Finally, for several risk factors (e.g., age,leukocytosis, thrombocytosis, and elevated ASO), relevant literature is limited and verification from clinical data is required. | 22 |
| Conclusions | 26 | In conclusion, our study highlights the following as factors associated with renal involvement in pediatric HSP: male gender, age > 10 years, severe gastrointestinal symptoms (including abdominal pain, gastrointestinal bleeding, and severe bowel angina), arthritis/arthralgia, persistent purpura or relapse, WBC > 15 × 10^9^/L, platelets > 500 × 10^9^/L, elevated ASO, and decreased C3. More attention should be paid to those children who have one or more of the above risk factors, and a sensitive scoring system based on these risk factors at onset may help predict renal injury in HSP patients. Relevant clinical intervention may exert positive effects on the prevention of kidney disease in the early stage of HSP. Well-designed and conventionally reported studies with a considerable number of children with HSP are necessary to determine the possible link between these 13 risk factors and HSPN. | 23 |
| **FUNDING** | | |  |
| Funding | 27 | This work was supported by the National Natural Science Foundation of China (Grant numbers 81270802, 81470946, 81200520) | 23-24 |

*From:*  Moher D, Liberati A, Tetzlaff J, Altman DG, The PRISMA Group (2009). Preferred Reporting Items for Systematic Reviews and Meta-Analyses: The PRISMA Statement. PLoS Med 6(7): e1000097. doi:10.1371/journal.pmed1000097

For more information, visit: **www.prisma-statement.org**.

Page 2 of 2
