# Supplementary figures and images for: Risk Factors Associated with Renal Involvement in Childhood Henoch-Schönlein Purpura: A Meta-Analysis
Source: PLoS One. 2016 Nov 30;11(11):e0167346. doi: 10.1371/journal.pone.0167346 (PMC5130272; doi:10.1371/journal.pone.0167346)

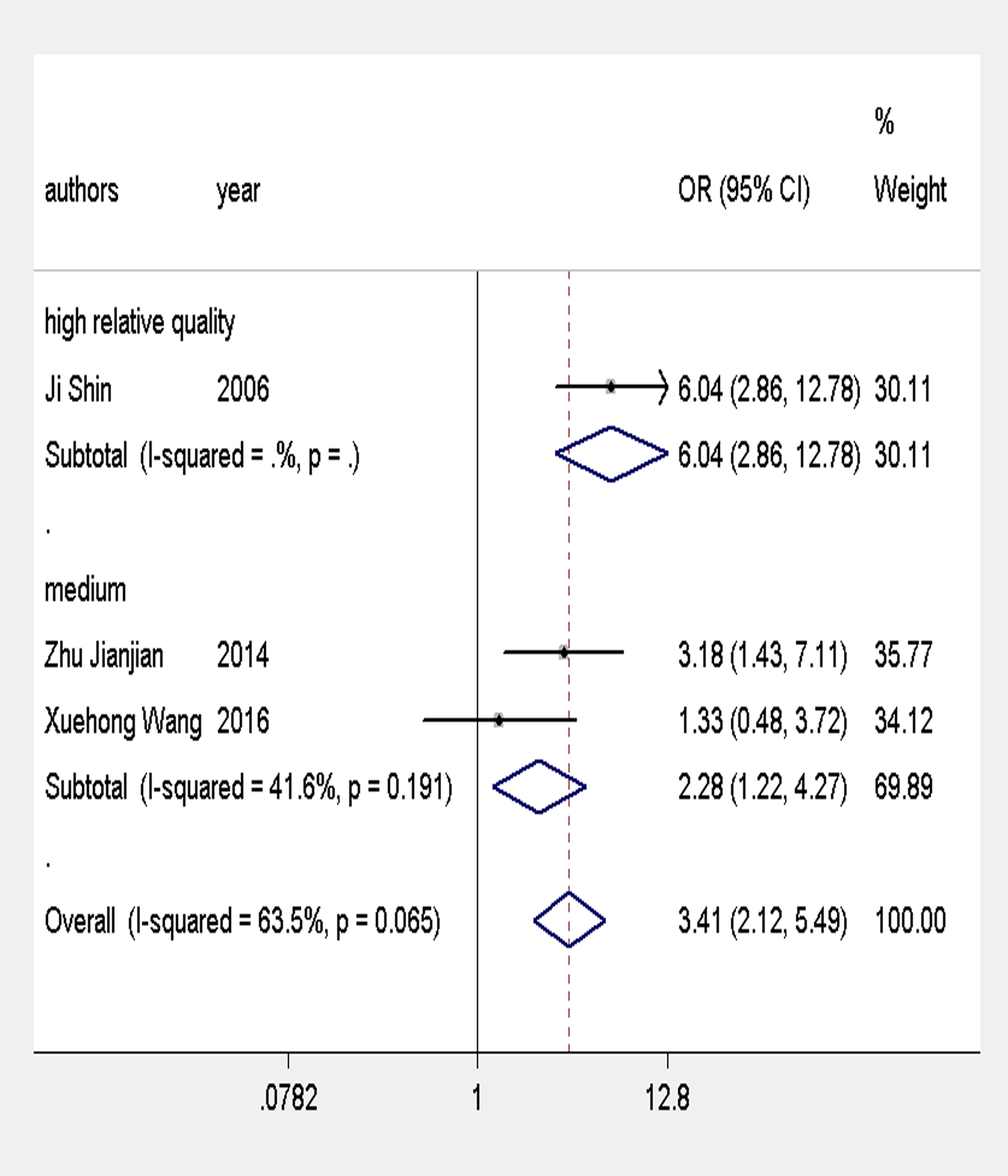

Supplement: S2 Appendix — Subgroup analysis for quality of evidence: (A) age; (B) male gender; (C) older age; (D) abdominal pain; (E) gastrointestinal bleeding; (F) severe bowel angina; (G) arthritis/arthralgia; (H) persistent purpura; (I) relapse; (J) leukocytosis; (K) thrombocytosis; (L) ASO; (M) C3 (ZIP) [file pone.0167346.s002.zip › S2 Appendix/S2 Appendix.(A)age.tif]

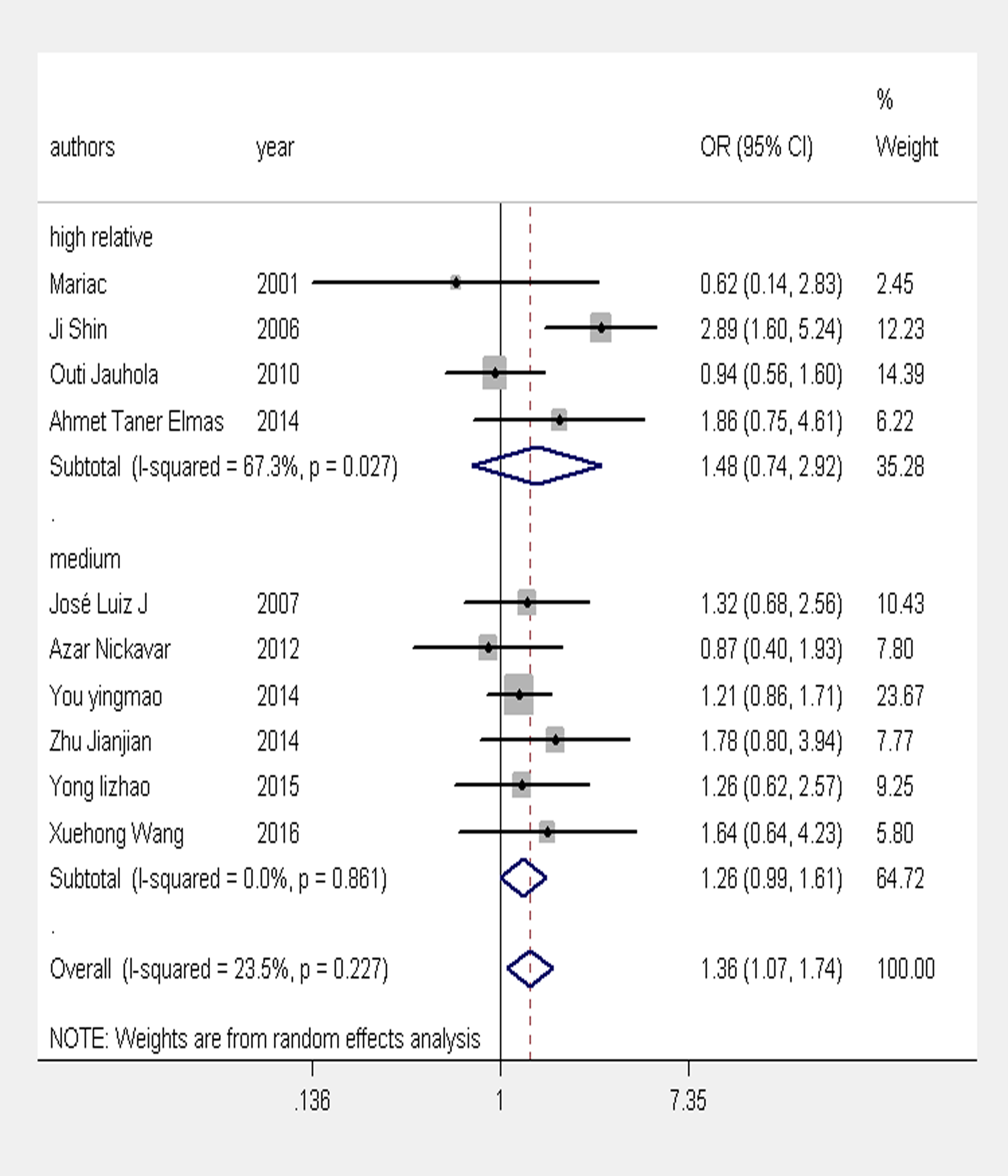

Supplement: S2 Appendix — Subgroup analysis for quality of evidence: (A) age; (B) male gender; (C) older age; (D) abdominal pain; (E) gastrointestinal bleeding; (F) severe bowel angina; (G) arthritis/arthralgia; (H) persistent purpura; (I) relapse; (J) leukocytosis; (K) thrombocytosis; (L) ASO; (M) C3 (ZIP) [file pone.0167346.s002.zip › S2 Appendix/S2 Appendix.(B)male gender.tif]

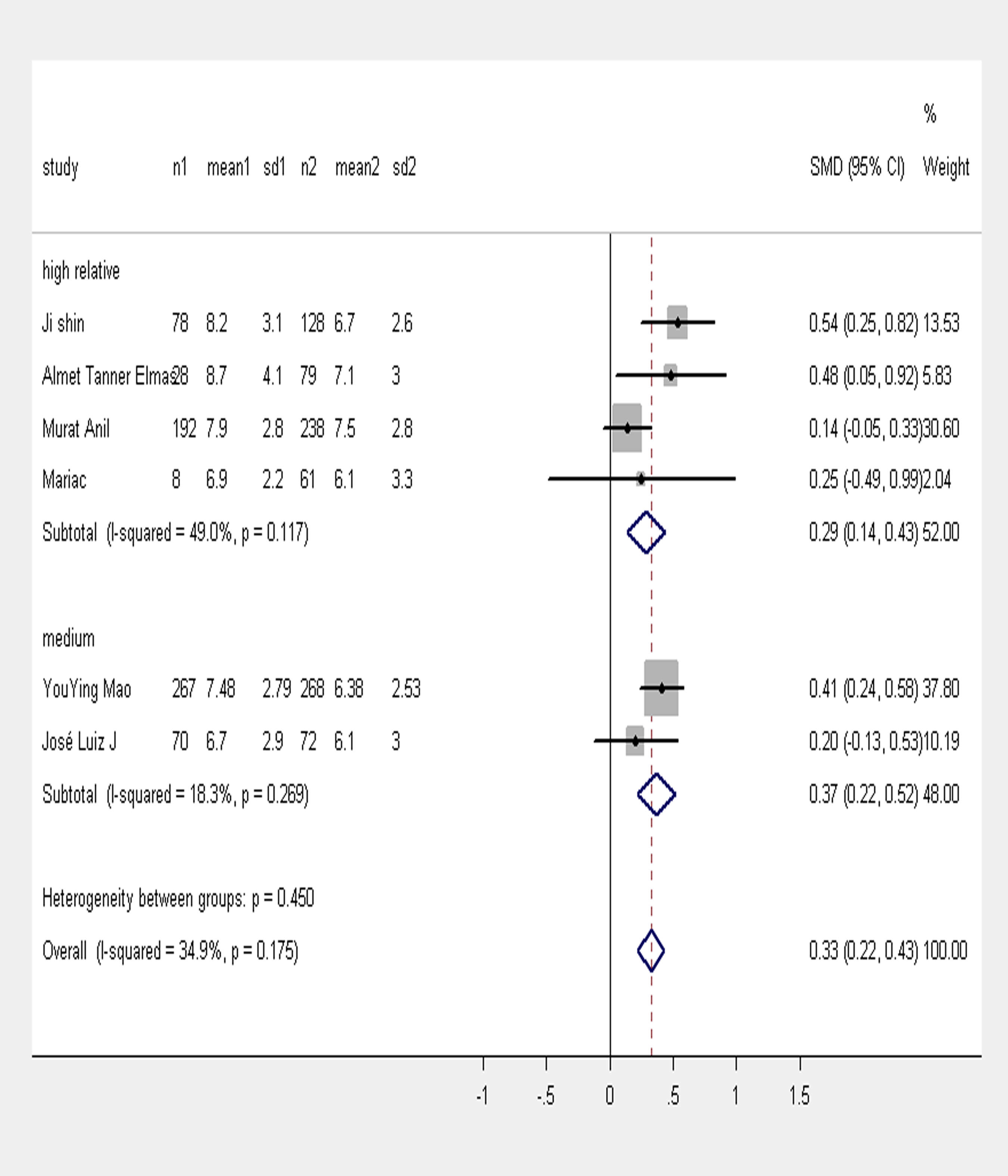

Supplement: S2 Appendix — Subgroup analysis for quality of evidence: (A) age; (B) male gender; (C) older age; (D) abdominal pain; (E) gastrointestinal bleeding; (F) severe bowel angina; (G) arthritis/arthralgia; (H) persistent purpura; (I) relapse; (J) leukocytosis; (K) thrombocytosis; (L) ASO; (M) C3 (ZIP) [file pone.0167346.s002.zip › S2 Appendix/S2 Appendix.(C)older age.tif]

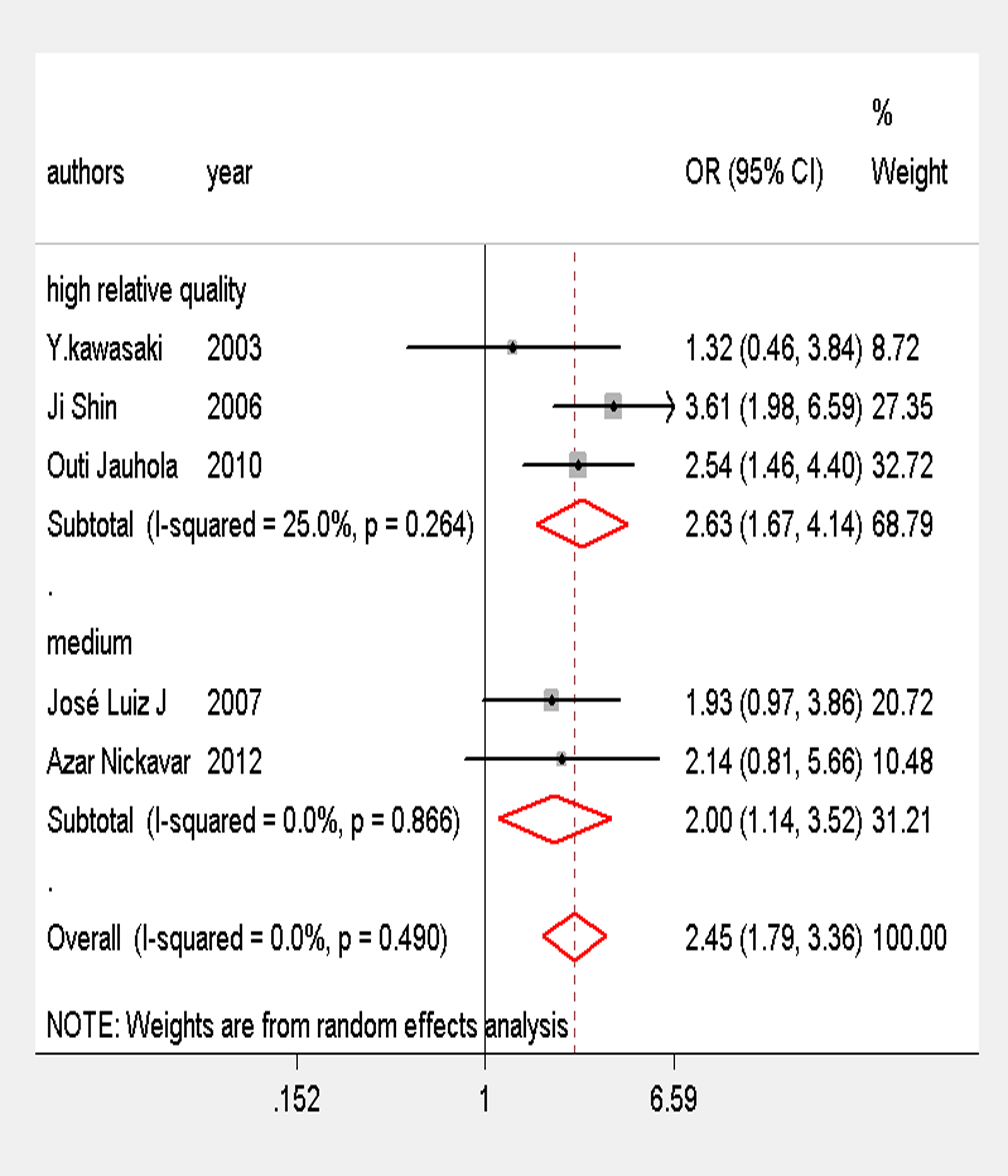

Supplement: S2 Appendix — Subgroup analysis for quality of evidence: (A) age; (B) male gender; (C) older age; (D) abdominal pain; (E) gastrointestinal bleeding; (F) severe bowel angina; (G) arthritis/arthralgia; (H) persistent purpura; (I) relapse; (J) leukocytosis; (K) thrombocytosis; (L) ASO; (M) C3 (ZIP) [file pone.0167346.s002.zip › S2 Appendix/S2 Appendix.(D)abdominal pain.tif]

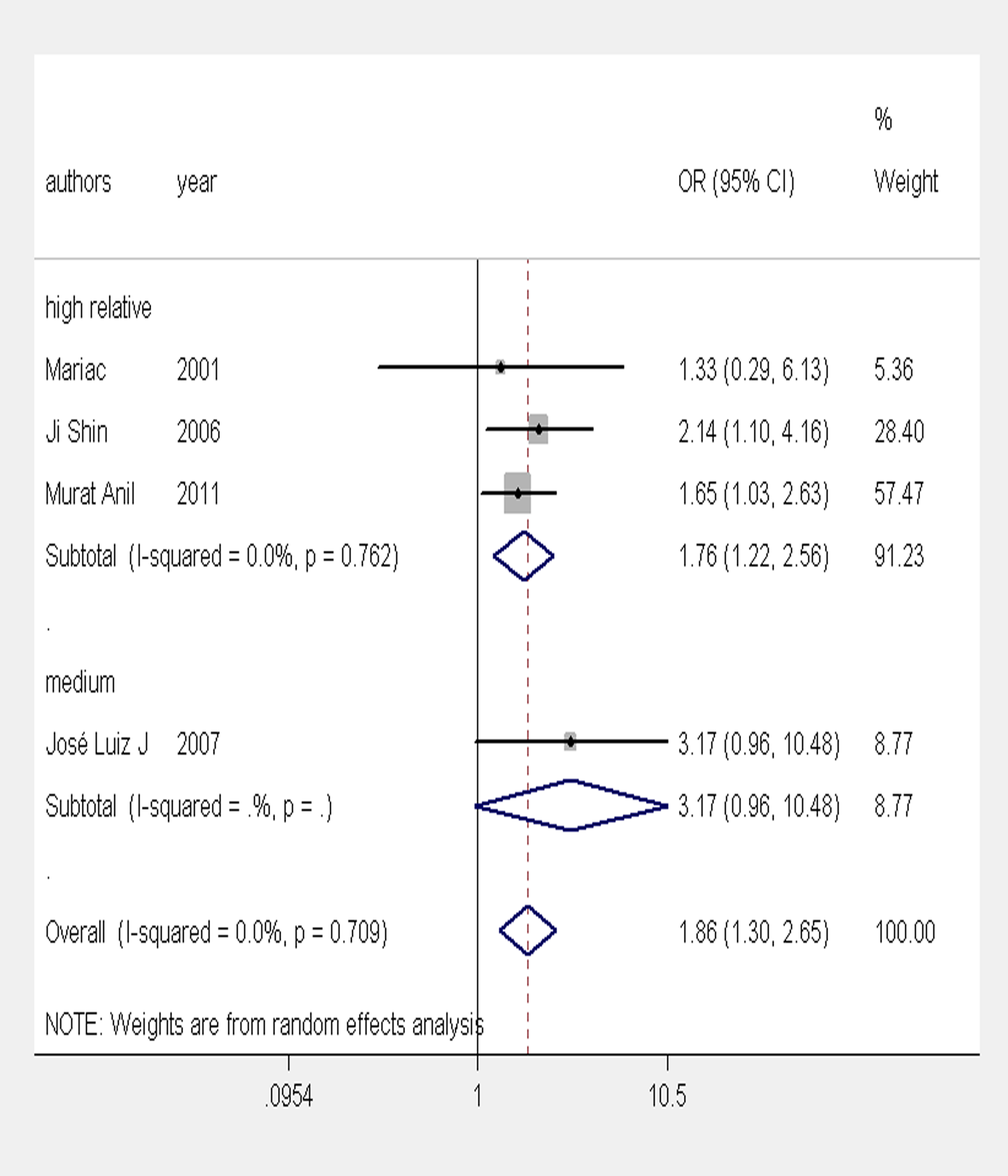

Supplement: S2 Appendix — Subgroup analysis for quality of evidence: (A) age; (B) male gender; (C) older age; (D) abdominal pain; (E) gastrointestinal bleeding; (F) severe bowel angina; (G) arthritis/arthralgia; (H) persistent purpura; (I) relapse; (J) leukocytosis; (K) thrombocytosis; (L) ASO; (M) C3 (ZIP) [file pone.0167346.s002.zip › S2 Appendix/S2 Appendix.(E)gastrointestinal bleeding.tif]

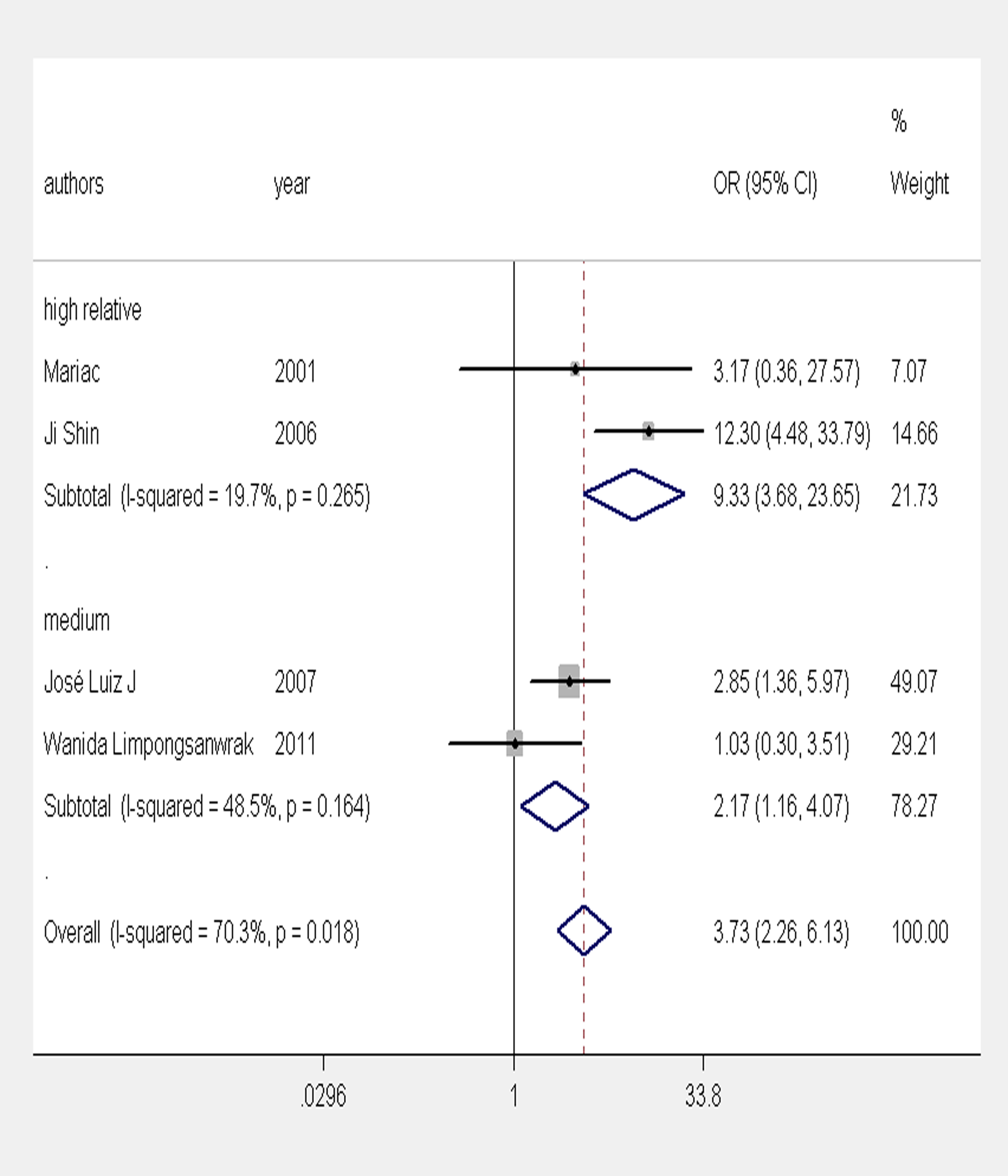

Supplement: S2 Appendix — Subgroup analysis for quality of evidence: (A) age; (B) male gender; (C) older age; (D) abdominal pain; (E) gastrointestinal bleeding; (F) severe bowel angina; (G) arthritis/arthralgia; (H) persistent purpura; (I) relapse; (J) leukocytosis; (K) thrombocytosis; (L) ASO; (M) C3 (ZIP) [file pone.0167346.s002.zip › S2 Appendix/S2 Appendix.(F)severe bowel angina.tif]

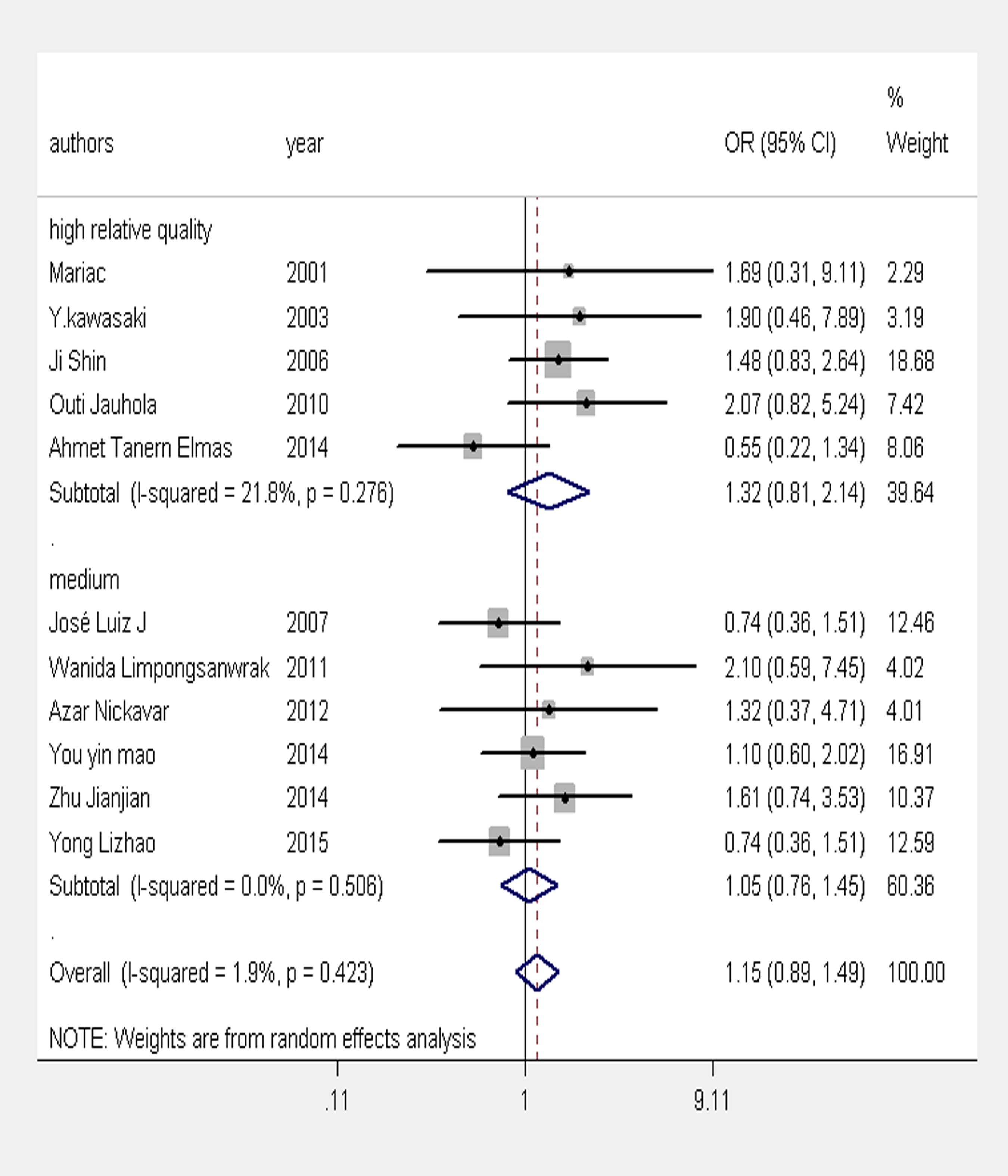

Supplement: S2 Appendix — Subgroup analysis for quality of evidence: (A) age; (B) male gender; (C) older age; (D) abdominal pain; (E) gastrointestinal bleeding; (F) severe bowel angina; (G) arthritis/arthralgia; (H) persistent purpura; (I) relapse; (J) leukocytosis; (K) thrombocytosis; (L) ASO; (M) C3 (ZIP) [file pone.0167346.s002.zip › S2 Appendix/S2 Appendix.(G)arthritis or arthralgia.tif]

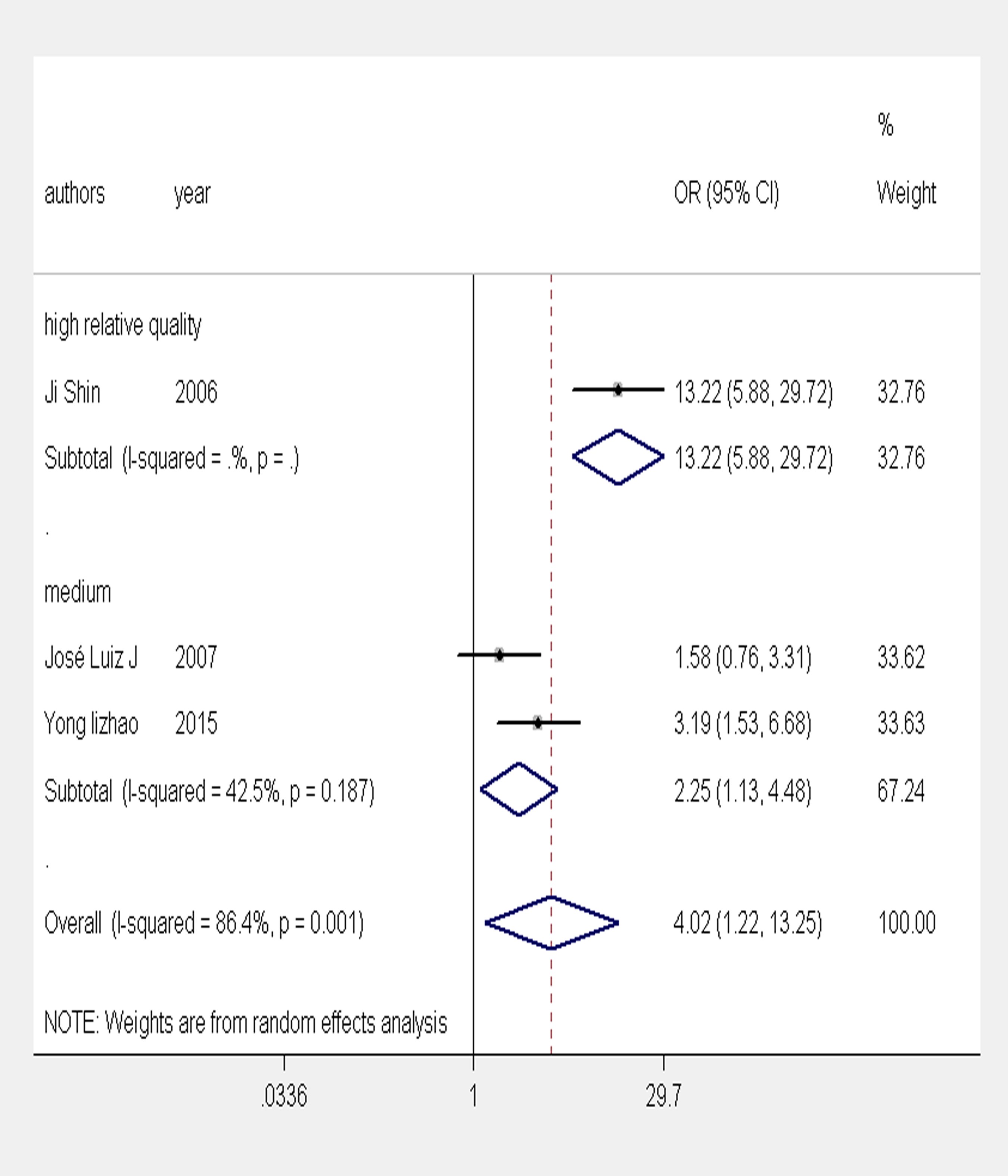

Supplement: S2 Appendix — Subgroup analysis for quality of evidence: (A) age; (B) male gender; (C) older age; (D) abdominal pain; (E) gastrointestinal bleeding; (F) severe bowel angina; (G) arthritis/arthralgia; (H) persistent purpura; (I) relapse; (J) leukocytosis; (K) thrombocytosis; (L) ASO; (M) C3 (ZIP) [file pone.0167346.s002.zip › S2 Appendix/S2 Appendix.(H)persistent purpura.tif]

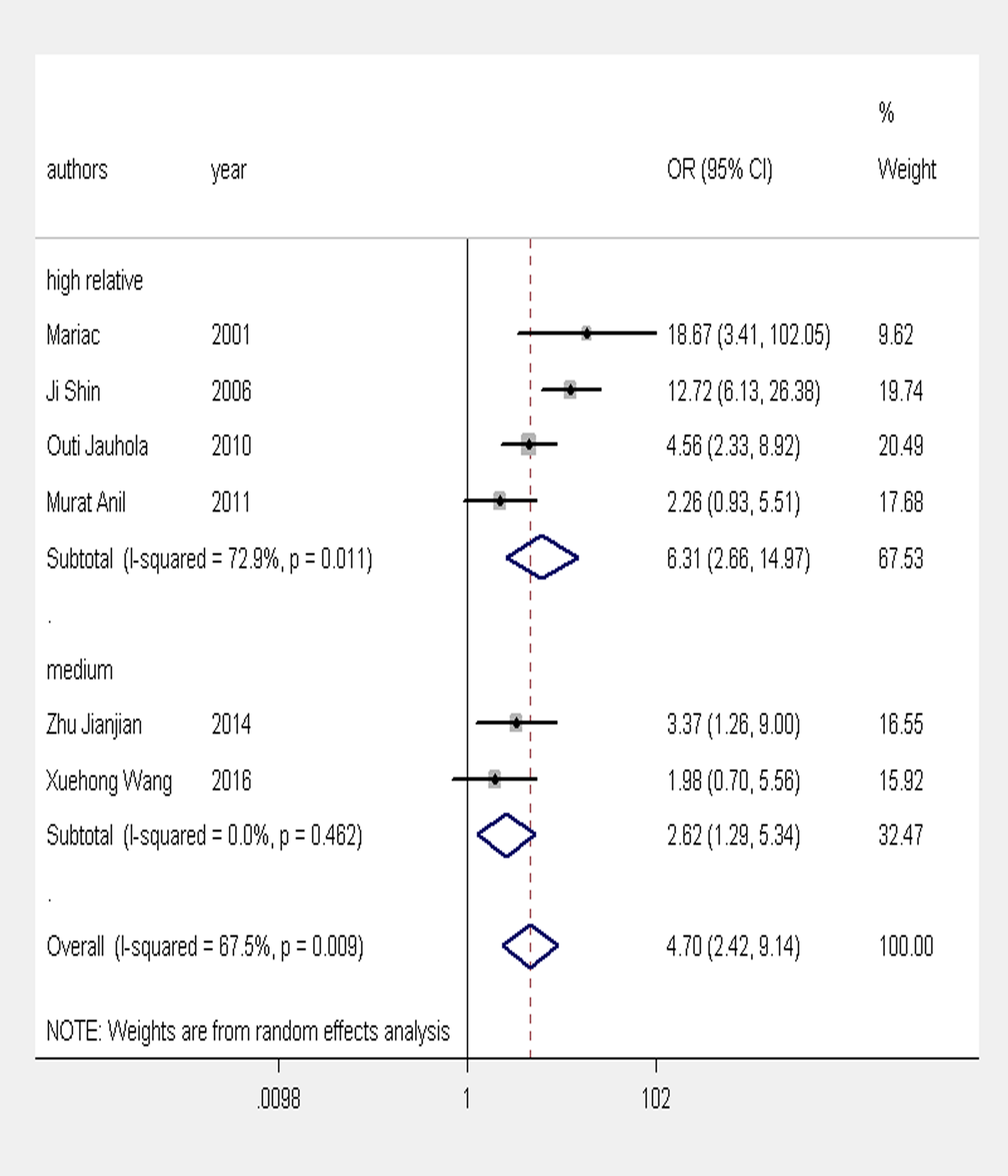

Supplement: S2 Appendix — Subgroup analysis for quality of evidence: (A) age; (B) male gender; (C) older age; (D) abdominal pain; (E) gastrointestinal bleeding; (F) severe bowel angina; (G) arthritis/arthralgia; (H) persistent purpura; (I) relapse; (J) leukocytosis; (K) thrombocytosis; (L) ASO; (M) C3 (ZIP) [file pone.0167346.s002.zip › S2 Appendix/S2 Appendix.(I)relapse.tif]

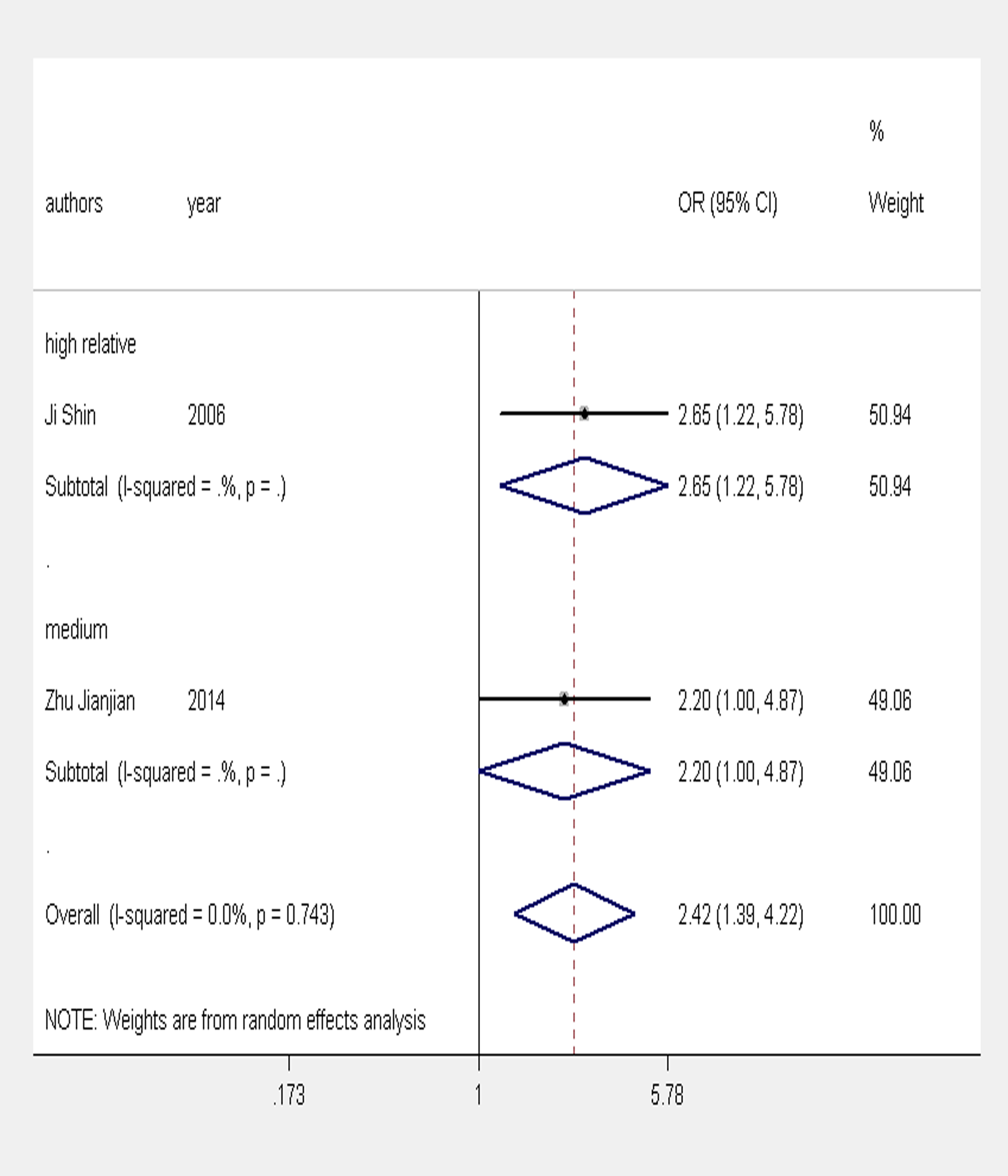

Supplement: S2 Appendix — Subgroup analysis for quality of evidence: (A) age; (B) male gender; (C) older age; (D) abdominal pain; (E) gastrointestinal bleeding; (F) severe bowel angina; (G) arthritis/arthralgia; (H) persistent purpura; (I) relapse; (J) leukocytosis; (K) thrombocytosis; (L) ASO; (M) C3 (ZIP) [file pone.0167346.s002.zip › S2 Appendix/S2 Appendix.(J)leukocytosis.tif]

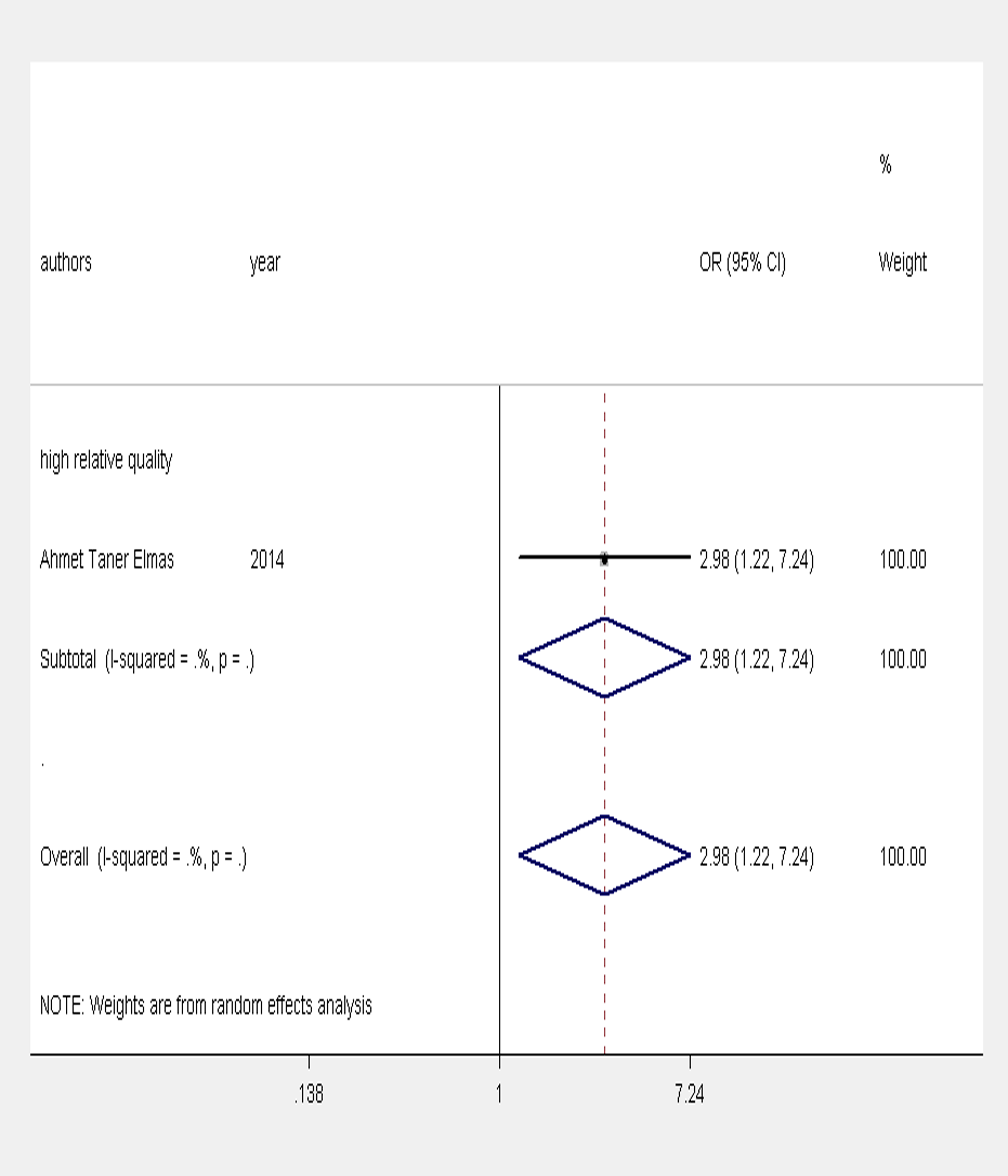

Supplement: S2 Appendix — Subgroup analysis for quality of evidence: (A) age; (B) male gender; (C) older age; (D) abdominal pain; (E) gastrointestinal bleeding; (F) severe bowel angina; (G) arthritis/arthralgia; (H) persistent purpura; (I) relapse; (J) leukocytosis; (K) thrombocytosis; (L) ASO; (M) C3 (ZIP) [file pone.0167346.s002.zip › S2 Appendix/S2 Appendix.(K)thrombocytosis.tif]

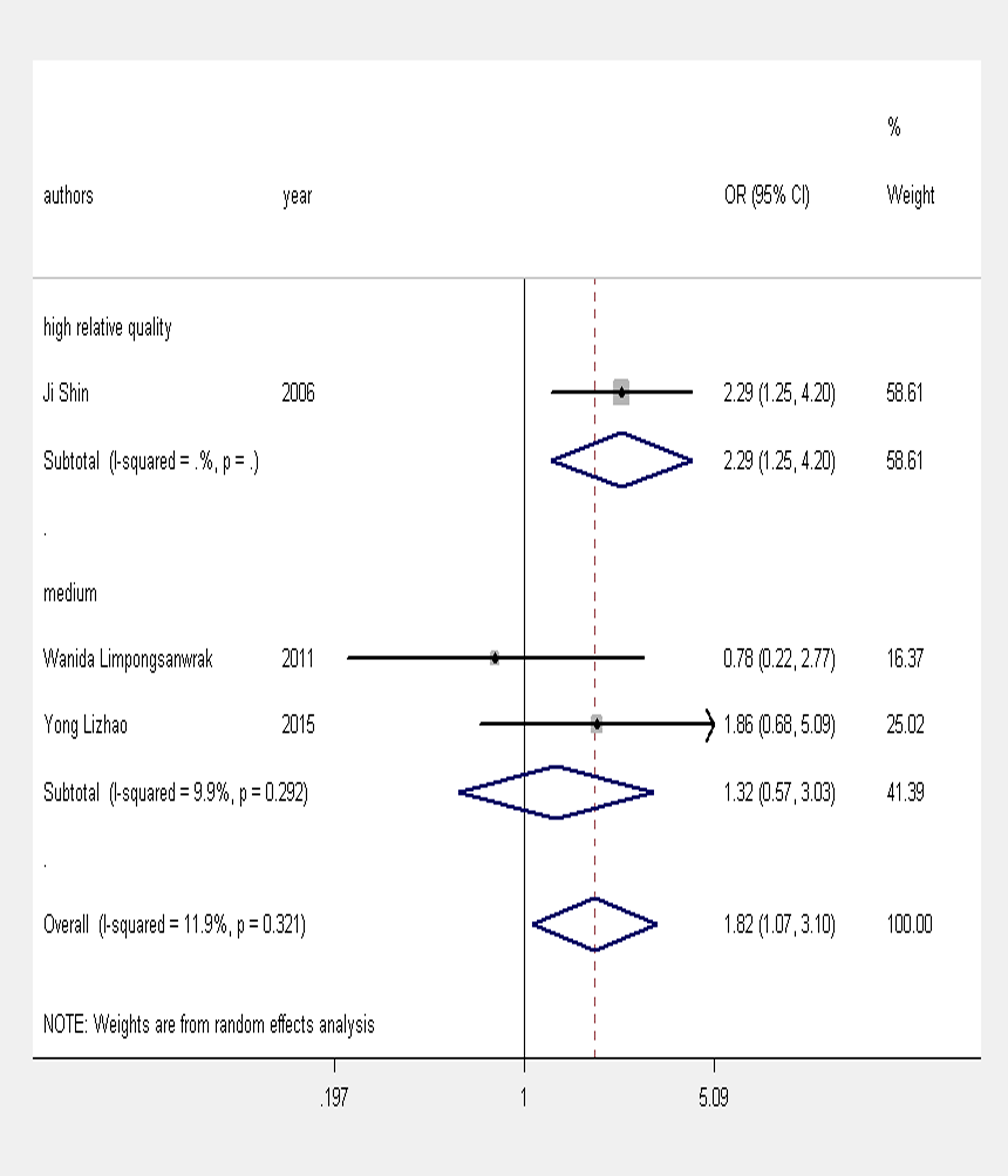

Supplement: S2 Appendix — Subgroup analysis for quality of evidence: (A) age; (B) male gender; (C) older age; (D) abdominal pain; (E) gastrointestinal bleeding; (F) severe bowel angina; (G) arthritis/arthralgia; (H) persistent purpura; (I) relapse; (J) leukocytosis; (K) thrombocytosis; (L) ASO; (M) C3 (ZIP) [file pone.0167346.s002.zip › S2 Appendix/S2 Appendix.(L)ASO.tif]

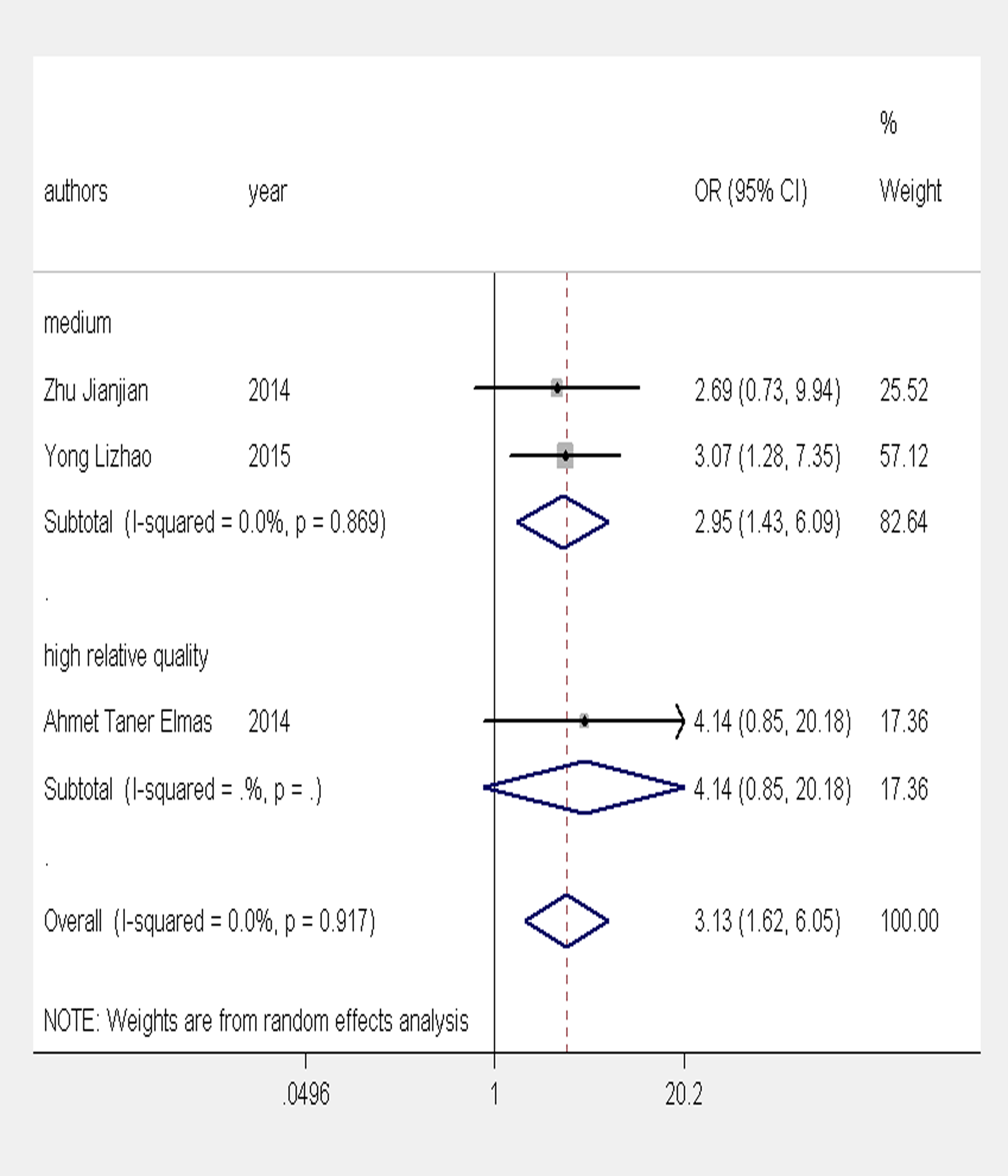

Supplement: S2 Appendix — Subgroup analysis for quality of evidence: (A) age; (B) male gender; (C) older age; (D) abdominal pain; (E) gastrointestinal bleeding; (F) severe bowel angina; (G) arthritis/arthralgia; (H) persistent purpura; (I) relapse; (J) leukocytosis; (K) thrombocytosis; (L) ASO; (M) C3 (ZIP) [file pone.0167346.s002.zip › S2 Appendix/S2 Appendix.(M)C3.tif]
