# Supplementary material for: Risk Factors Associated with Renal Involvement in Childhood Henoch-Schönlein Purpura: A Meta-Analysis
Source: PLoS One. 2016 Nov 30;11(11):e0167346. doi: 10.1371/journal.pone.0167346 (PMC5130272; doi:10.1371/journal.pone.0167346)
Supplement: S1 Text — (DOC) [file pone.0167346.s003.doc]

**PubMed:**

#1 "purpura, schoenlein-henoch"[MeSH Terms]

#2 Henoch-Schönlein purpura [All Fields]

#3 #1 OR #2

#4 "Risk Factors"[Mesh]

#5 risk factors [All Fields]

#6 #4 OR #5

#7 #3 AND #6

#8 Filters: Publication date from 2000/01/01 to 2016/09/0**1**

#9 #7 AND #8

**Embase:**

#1 'anaphylactoid purpura'/exp

#2 henoch-schönlein purpura

#3 #1 OR #2

#4 'risk factor'/exp

#5 #3 AND #4

#6 [1-1-2000]/sd NOT [1-9-2016]/sd

#7 #5 AND #6

**Web of Science**

#1 TS="Henoch-Schönlein purpura"

#2 TS="risk factor"

#3 TS="risk factors"

#4 #3 OR #2

#5 #4 AND #1

#6 PY=2000-2016

#7 #5AND #6
